# MANNA-VISW: A Low-Cost Multimodal Autonomous Platform for Environmental and Ecosystem Recovery Monitoring in Disconnected Post-Disaster Scenarios

**DOI:** 10.3390/s26175522

**Published:** 2026-08-31

**Authors:** André B. Verona, Hani C. Yehia, Geraldo Wilson Fernandes, Yumi Oki, Linnyer B. R. Aylon

**Affiliations:** 1 Manna Research Group, Universidade Federal de Minas Gerais, Belo Horizonte 31270-901, Brazil; lbruiz@uem.br; 2 Manna Research Group, Universidade Estadual de Maringá, Maringá 87020-900, Brazil; 3 Graduate Program in Electrical Engineering, Universidade Federal de Minas Gerais, Belo Horizonte 31270-901, Brazil; hani@cpdee.ufmg.br; 4 Department of Electronic Engineering, Universidade Federal de Minas Gerais, Belo Horizonte 31270-901, Brazil; 5 Department of Genetics, Ecology and Evolution (DGEE), Institute of Biological Sciences (ICB), Universidade Federal de Minas Gerais (UFMG), Belo Horizonte 31270-901, Brazil; gwfernandes@ufmg.br (G.W.F.); yumioki@ufmg.br (Y.O.)

**Keywords:** environmental sensing, IoT, multimodal environmental monitoring, ecoacoustics, passive acoustic monitoring, ecosystem recovery, phenological monitoring, post-disaster monitoring

## Abstract

Monitoring environmental degradation requires robust solutions capable of capturing the complex dynamics of ecosystems. This study presents VISW (Verona Image, Sound, and Weather), a low-cost autonomous platform designed for long-term operation in remote areas, developed with support from the MANNA and LEEB research groups. The system employs a multimodal sensing approach, integrating weather data acquisition, multispectral imaging, and environmental acoustic monitoring. VISW was engineered for continuous autonomous operation, incorporating dedicated energy management, data acquisition, and storage subsystems suitable for adverse field conditions. This work was developed in the context of the Fundão dam collapse (2015), one of the largest environmental disasters in Brazil’s history. To monitor biodiversity recovery in the impacted areas, nine VISW Station units were deployed throughout the Doce River basin (Minas Gerais, Brazil). Data collection has been continuously carried out since September 2024, creating a valuable multimodal dataset composed of environmental sounds, multispectral images, and meteorological data from the affected regions. This dataset enables the investigation of complex relationships among vegetation dynamics, climatic patterns, and local soundscapes, providing a comprehensive understanding of ecological processes in areas impacted by disasters. Finally, field tests validated the VISW as a robust, scalable, and cost-effective solution for long-term environmental monitoring.

## 1. Introduction

Environmental monitoring has significantly advanced in recent decades, driven by the rapid development of low-cost sensors, embedded systems, and wireless communication technologies. These advances have enabled the deployment of distributed monitoring systems capable of collecting environmental data in real time, supporting applications ranging from precision agriculture to climate analysis [1,2,3].

Traditional Internet of Things (IoT) frameworks designed for environmental sensing often rely on persistent cloud connectivity for data processing and transmission. These frameworks simply cannot be used in disconnected remote or post-disaster environments where communication infrastructures may be severely compromised or entirely absent. To bridge this gap, this paper introduces MANNA-VISW (Verona Image, Sound, and Weather platform, developed by the Manna Research Group), an autonomous, low-cost multimodal edge platform designed to operate in entirely disconnected environments. We demonstrate the viability of this approach by analyzing a comprehensive multimodal dataset collected in situ over an eighteen-month deployment.

One of the most significant environmental disasters in Brazil occurred on 5 November 2015, with the collapse of the Fundão tailings dam in Mariana, Minas Gerais. The disaster released approximately 55–62 million cubic meters of mining waste along the Doce River basin, causing extensive environmental degradation and severe impacts on local ecosystems [4,5]. Previous studies have primarily focused on isolated aspects of the impacted environment, such as soil contamination and water quality, reinforcing the need for continuous and integrated environmental monitoring approaches capable of long-term autonomous operation under limited connectivity conditions.

Recent research has increasingly emphasized the adoption of autonomous edge-based architectures for environmental monitoring applications. By shifting computational and storage tasks directly to the deployment site, low-cost embedded nodes can process heterogeneous sensor streams locally and operate entirely offline [6].

This approach enables multi-month and multi-year monitoring protocols, supporting the continuous monitoring of environmental indicators associated with ecosystem degradation and ecological recovery in highly impacted mining areas [7].

Edge computing architectures have been increasingly adopted in environmental monitoring systems to enable local data processing, reduce latency, and improve operational autonomy in scenarios with limited connectivity [8].

Ecoacoustic approaches have emerged as powerful tools for biodiversity and ecosystem monitoring. Passive Acoustic Monitoring (PAM) enables continuous and non-invasive data acquisition, allowing the characterization of soundscapes as proxies for ecological processes [9,10,11]. Autonomous acoustic recording systems have been widely used for long-term environmental monitoring, particularly in remote areas where traditional methods are limited. Towsey demonstrated that large-scale acoustic datasets can be used to identify temporal and spatial ecological patterns, highlighting the potential of long-duration recordings for ecosystem analysis [12]. Scarpelli et al. demonstrated that variations in soundscape composition are strongly associated with landscape structure and vegetation conditions in Atlantic Forest environments, reinforcing the use of acoustic data as indicators of environmental quality [13].

Remote sensing and image-based techniques have also become increasingly important for evaluating vegetation dynamics and ecosystem responses to environmental changes. Continuous near-surface remote sensing initiatives, such as the PhenoCam network [14], have demonstrated the potential of automated digital imaging systems for long-term phenological and ecosystem monitoring. Recent studies using phenocameras in tropical ecosystems have also demonstrated strong relationships between climatic variability, vegetation phenology, and ecosystem productivity [15]. Vegetation indices, such as the Normalized Difference Vegetation Index (NDVI), have been widely used to monitor ecosystem responses to environmental changes, providing valuable information about plant health and biomass [16,17].

The integration of multiple environmental data sources has expanded the capability of monitoring systems to capture complex ecological interactions. Multimodal approaches combining climatic, acoustic, and visual data have shown promising results in capturing complex ecological interactions and improving the interpretation of ecosystem dynamics [18,19]. The integration of heterogeneous environmental data enables the correlation of different environmental variables, allowing the identification of ecological patterns that are difficult to observe using a single sensing modality [18].

Many existing environmental monitoring approaches focus on single data modalities, reducing their ability to capture the complex interactions between biotic and abiotic ecosystem components. This limitation becomes particularly critical in post-disaster scenarios.

This work proposes a multimodal environmental monitoring system capable of operating in remote areas without communication infrastructure. The system integrates climatic sensors, acoustic monitoring, and dual-band imaging within an edge-enable computing architecture, enabling synchronized acquisition of heterogeneous environmental data.

The VISW Station platform was developed with support from the MANNA Research Group at the Universidade Estadual de Maringá (UEM) and the LEEB Research Group at the Universidade Federal de Minas Gerais (UFMG).

To validate the operational capability, energy autonomy, and multimodal data synchronization of the proposed system under real-world conditions, data collected during a continuous deployment period from September 2024 to March 2026 were analyzed as a representative case study.

Environmental monitoring platforms have been proposed in recent years to address specific aspects of ecosystem monitoring, including weather sensing, passive acoustic monitoring, phenological imaging, and multimodal IoT architectures. A comparison of selected studies from the literature and their main characteristics relative to the proposed VISW Station is presented in Table 1.

## 2. Materials and Methods

### 2.1. Study Area

The environmental monitoring stations were installed along the Gualaxo do Norte and Doce River Basins, located in Minas Gerais, Brazil, a region severely impacted by the Fundão dam collapse in 2015. The disaster released approximately 55–62 million cubic meters of mining tailings, causing extensive environmental damage along the Doce River basin and severely affecting local ecosystems [4,20].

The monitoring network consists of nine autonomous environmental monitoring stations distributed across regions presenting different vegetation structures, climatic conditions, and levels of environmental recovery. The monitored areas include regions with preserved vegetation, regenerating ecosystems, urban influence, and regions directly affected by the mining disaster.

**Figure 1:**
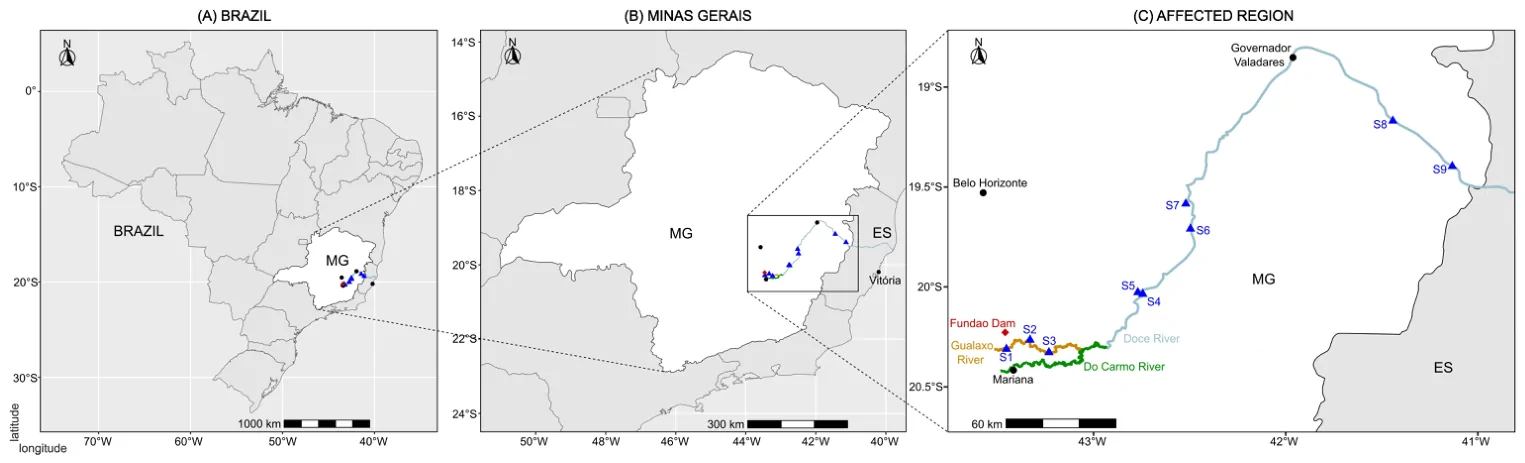
Geographic distribution of the environmental monitoring stations installed along the Gualaxo do Norte (orange), do Carmo (green) and Doce River (blue) basins in Minas Gerais, Brazil: (**A**) Brazil; (**B**) Minas Gerais State; and (**C**) detailed view of the study area and monitoring station locations.

To provide a more detailed visualization of the study areas, Figure 1 presents the geographic distribution of the monitoring stations, while Table 2 summarizes the coordinates and sampling locations. The monitoring network covers an approximate spatial extent of 9600 km^2^, considering the convex area delimited by the nine VISW Station deployment sites along the Gualaxo do Norte and Doce River basins.

**Figure 2:**
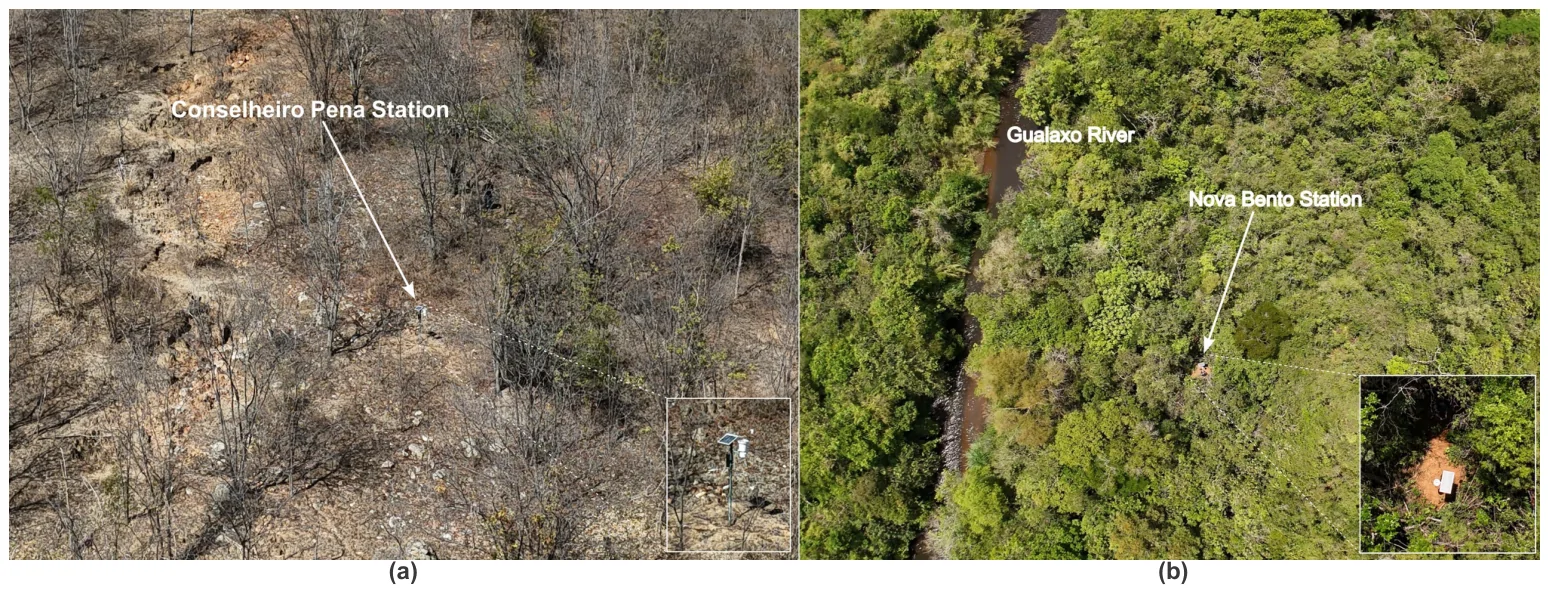
Aerial drone images of representative monitoring locations at Conselheiro Pena (**a**) and Nova Bento Rodrigues (**b**), illustrating differences in vegetation structure, terrain characteristics and the deployment of the VISW monitoring stations.

Additionally, the monitoring stations were installed under distinct environmental and topographic conditions, including riparian vegetation, secondary forest fragments and regions undergoing ecological regeneration after the mining disaster. Figure 2 presents aerial drone images of two representative monitoring locations, highlighting differences in vegetation density, terrain characteristics, and surrounding environmental conditions between the Nova Bento Rodrigues and Conselheiro Pena stations.

### 2.2. VISW Station Architecture

The proposed environmental monitoring station was designed as a low-cost autonomous platform capable of long-term operation in remote environments without communication infrastructure.

Each station integrates climatic, acoustic, and visual sensing modules connected to a Raspberry Pi Zero 2 W embedded computer [21]. This platform was selected due to its balance between computational capability, low power consumption, and hardware flexibility for multimodal environmental sensing applications. The platform provides a quad-core Advanced RISC (Reduced Instruction Set Computer) Machine (ARM) Cortex-A53 processor operating at 1 GHz and 512 MB of Random Access Memory (RAM), enabling future edge-computing applications and local environmental data processing. In addition, the native Camera Serial Interface (CSI) supports high-resolution Red, Green and Blue (RGB) and No Infrared Filter (NOIR) camera modules while maintaining compatibility with Linux-based software environments and Python (version 3.9.2) automation scripts used for synchronized multimodal data acquisition.

Climatic monitoring was performed using commercially available low-cost electronic modules acquired from Chinese suppliers, using DHT22 (AM2302) sensors, for air temperature and relative humidity measurements, DS18B20 sensors for soil temperature acquisition, and tipping-bucket rain gauges for rainfall estimation.

Environmental acoustic acquisition was performed using an INMP441 digital microphone connected through the Inter-Integrated Circuit Sound (I2S) interface of the Raspberry Pi Zero 2 W platform. The microphone used General Purpose Input/Output (GPIO) 18 for serial clock (SCK), GPIO 19 for Word Select (WS), and GPIO 20 for Serial Data (SD) acquisition.

Visual monitoring was performed using a single OV5647 5 MP camera module equipped with a mechanically switchable infrared filter. The filter position was controlled through an L298N H-bridge module by alternating the polarity between +3.3 V and −3.3 V, enabling sequential acquisition of conventional RGB and NOIR images using the same imaging sensor.

Energy autonomy was achieved using photovoltaic panels, lithium battery packs, and charge controller modules. Depending on local environmental conditions, stations installed in areas with continuous solar exposure employed 10 W photovoltaic panels, while stations located near dense vegetation or partially shaded environments used 30 W panels to compensate for reduced solar irradiance.

Energy storage was provided by 3S 11.1 V lithium-ion battery packs based on 18,650 cells with a total capacity of 15 Ah. This battery configuration was selected due to its high energy density, compact physical dimensions, and suitability for installation inside slim hermetic enclosures used in the proposed environmental monitoring architecture.

After system initialization, the environmental monitoring station presented an average current consumption of approximately 120 mA under the 12 V power supply system, corresponding to an average power consumption close to 1.44 W. Short-duration current peaks of up to 400 mA were observed during image acquisition events due to camera activation and infrared filter switching operations.

Considering the adopted 11.1 V battery configuration with a total capacity of 15 Ah, the system provided approximately 166.5 Wh of available energy. Under theoretical conditions, the adopted battery configuration would therefore be capable of maintaining autonomous station operation for approximately 4.8 days without solar energy input.

The adopted 10 W photovoltaic panels, operating with nominal voltages around 18 V and current levels between 0.5 and 0.6 A under strong solar irradiance conditions, were theoretically capable of supplying the station energy demand with approximately 3.5 to 4 h of effective peak solar exposure per day.

**Figure 3:**
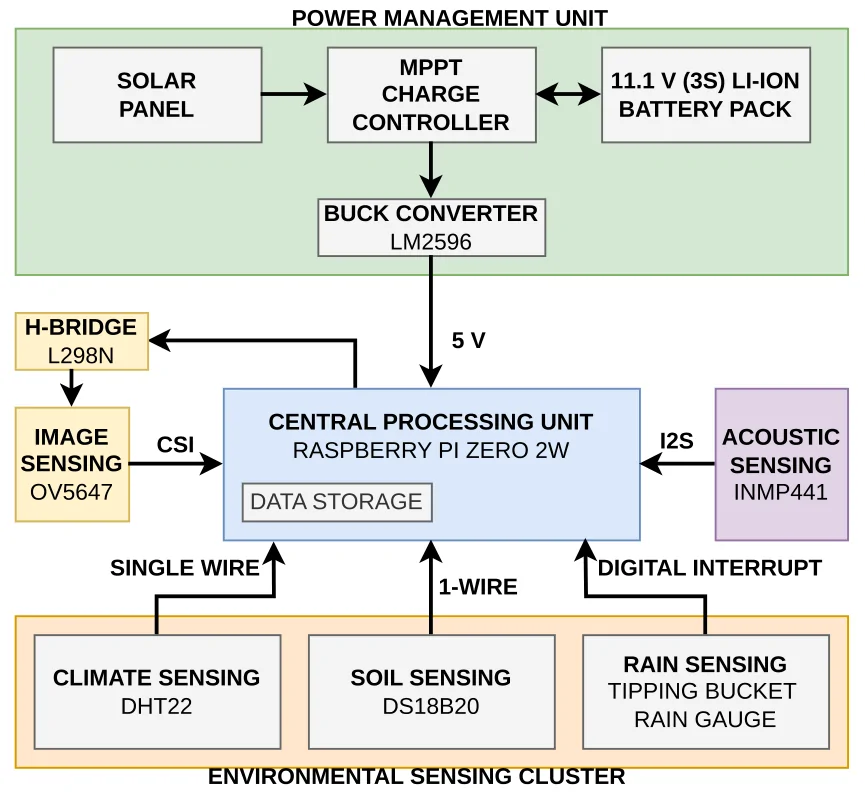
Block diagram of the proposed VISW Station architecture for multimodal environmental monitoring, including power management, sensing modules, and embedded processing.

The proposed VISW Station multimodal environmental monitoring architecture is illustrated in Figure 3. The system integrates climatic, acoustic, and image-based sensing modules into a low-cost autonomous platform centered on a Raspberry Pi Zero 2 W embedded computer.

The physical structure of the monitoring station was designed to improve long-term outdoor reliability under adverse environmental conditions. The imaging sensor was installed below the photovoltaic panel in order to reduce direct exposure to rainfall and excessive solar radiation while maintaining an unobstructed field of view. The tipping-bucket rain gauge was laterally displaced using a support arm to minimize interference caused by water runoff from the solar panel surface. Shielded cables and hermetically sealed enclosures were employed to improve protection against humidity, rain, dust, and electromagnetic interference.

The upper sealed enclosure houses the camera and acoustic sensing modules, while the larger lower enclosure contains the battery pack, Maximum Power Point Tracking (MPPT) charge controller, power regulation circuitry, and processing electronics. This modular arrangement facilitates maintenance procedures and improves environmental protection of critical electronic components during continuous field operation.

The physical deployment structure was designed to enable rapid installation under diverse environmental conditions while maintaining low cost and mechanical simplicity. The monitoring stations were mounted using galvanized steel square tubes (30 mm × 30 mm, 1.2 mm wall thickness). Initially, a short guide tube was temporarily inserted into the soil to create the pilot hole. After removal of the guide structure, the main station support tube, approximately 2.2 m in height, was inserted and fixed into the ground to a depth of approximately 40 cm.

This deployment strategy enabled rapid field installation within a few minutes while maintaining sufficient mechanical stability for long-term environmental monitoring. The adopted structure also allowed installation in locations with irregular topography and steep terrain, including slopes and embankments, significantly expanding the potential deployment scenarios for ecological monitoring applications. The galvanized steel structure also improved corrosion resistance during prolonged outdoor deployment under humid tropical environmental conditions.

Additional external resistors were employed in the sensor interface circuitry in order to improve signal stability and reliability during long-term outdoor operation. The DHT22 climatic sensor utilized a 10 kΩ pull-up resistor connected between the data line and the 3.3 V supply voltage, ensuring stable digital communication with the Raspberry Pi GPIO interface. Similarly, the DS18B20 soil temperature sensor employed a 4.7 kΩ pull-up resistor in the 1-Wire communication line, following the standard electrical configuration recommended for reliable OneWire communication protocols.

For the tipping-bucket rain gauge interface, a 4.7 kΩ pull-down resistor was connected between the GPIO input and ground in order to stabilize the digital signal and minimize false triggering caused by electrical noise or long cable interference under field conditions. The rain gauge output was connected to Raspberry Pi GPIO pin 26 and configured for interrupt-based pulse detection using rising-edge transitions.

Rainfall acquisition employed hardware interrupt detection combined with software debounce filtering in order to reduce false pulse generation caused by mechanical bouncing effects of the tipping-bucket mechanism. A debounce interval of 200 ms was implemented in the acquisition software, preventing multiple interrupt detections during a single tipping event and improving rainfall measurement reliability during continuous long-term operation.

Table 3 summarizes the main GPIO connections and sensing interfaces employed in the proposed environmental monitoring station.

**Figure 4:**
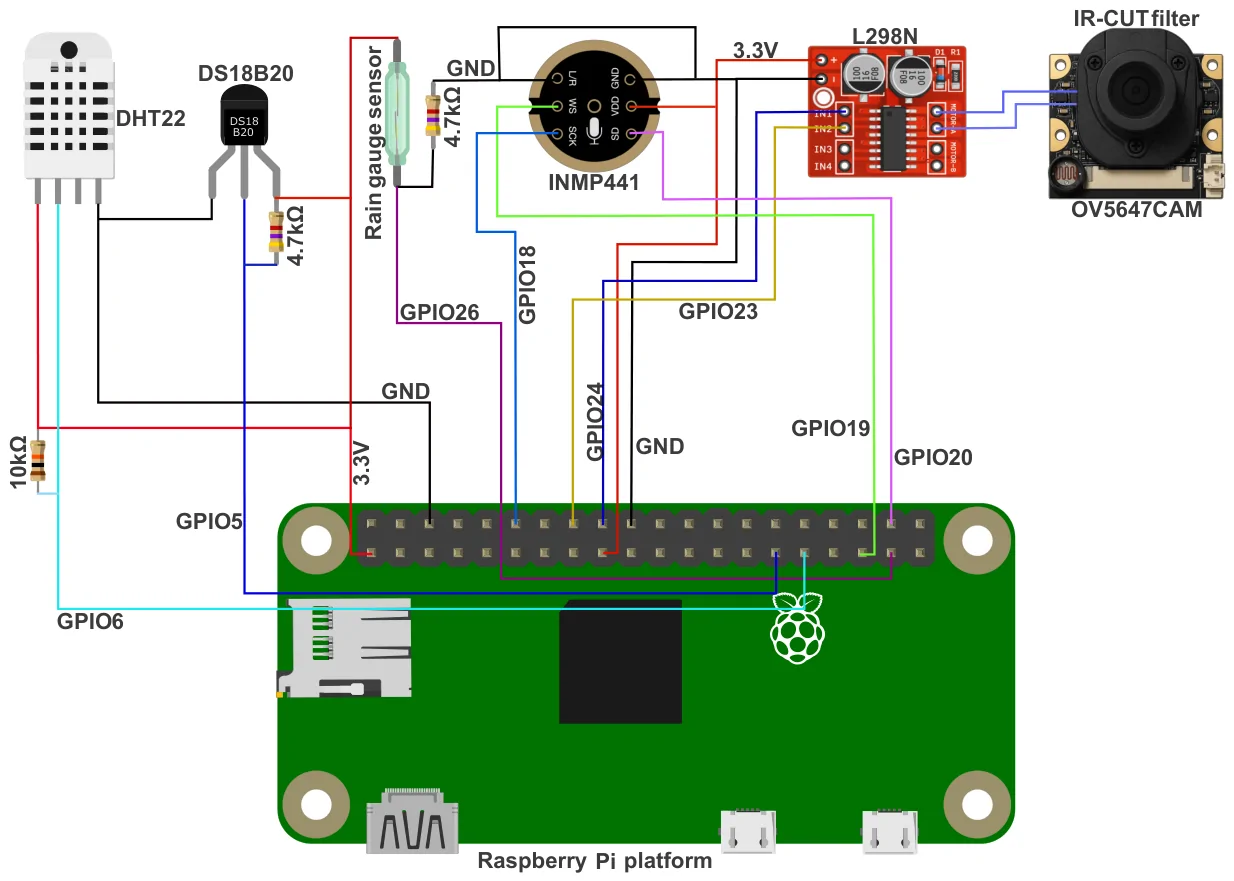
Electrical circuitry and GPIO connections employed in the VISW environmental monitoring station, including climatic sensors, rainfall acquisition system, acoustic monitoring interface, and IR-CUT filter actuation circuitry.

The GPIO electrical interfaces illustrated in Figure 4 are employed in the environmental monitoring station, including climatic sensing modules, rainfall pulse acquisition circuitry, acoustic monitoring interfaces, and the infrared-cut (IR-CUT) filter actuation system.

Image acquisition was performed using a modified OV5647 camera module connected through the dedicated MIPI CSI-2 interface of the Raspberry Pi Zero 2 W platform, allowing high-speed image transfer while preserving GPIO resources for environmental sensing and peripheral control tasks. By default, the adopted camera module employs an onboard Light-Dependent Resistor (LDR) responsible for automatically activating the mechanical IR-CUT filter during low-light conditions. The automatic switching mechanism was intentionally disabled to enable direct software-based control of the IR-CUT filter through the proposed L298N H-bridge circuitry. This approach enabled controlled and repeatable acquisition of paired RGB and NOIR images under identical environmental conditions.

**Figure 5:**
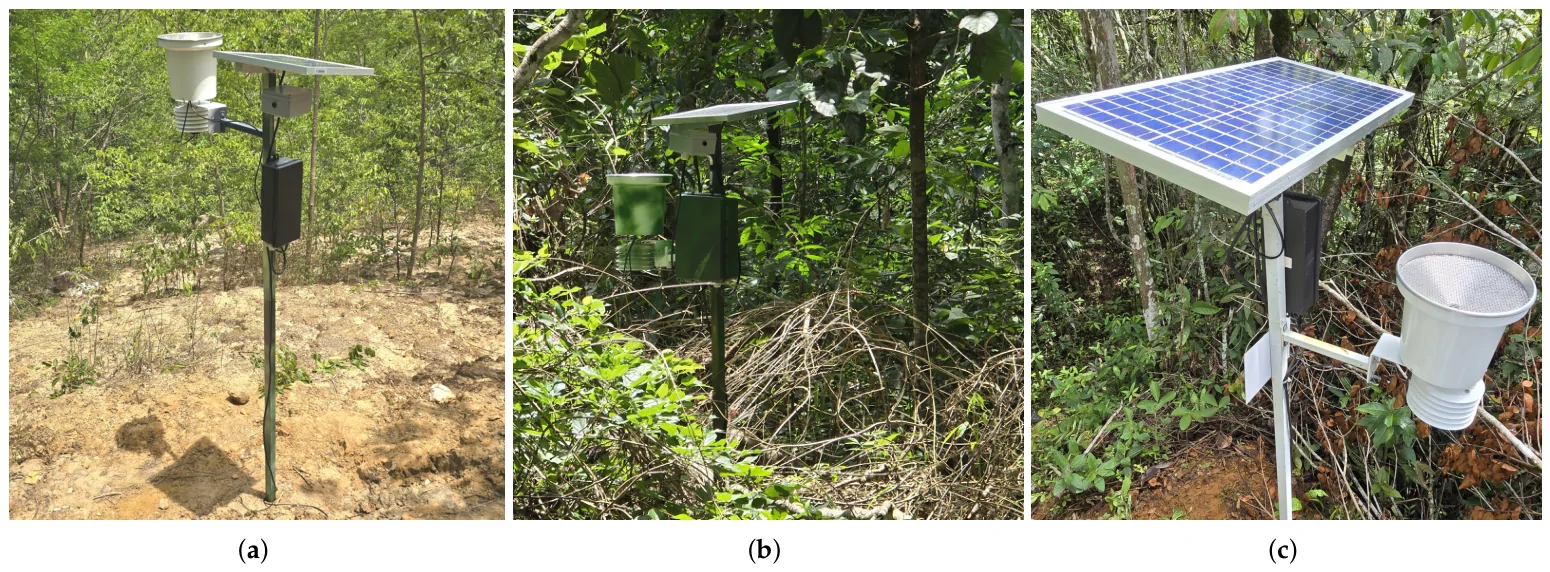
Examples of deployed VISW environmental monitoring stations installed in different monitoring regions: Conselheiro Pena (**a**), Ipatinga 1 (**b**), and Nova Bento Rodrigues (**c**).

Figure 5 also shows the central support structure, the solar panel positioned above the system to protect the hermetic enclosures from direct sunlight and rain exposure, and the rain gauge installed laterally using an extension arm to avoid interference from water runoff interacting with the solar panel.

#### Bill of Materials (BOM) of the VISW Station

To provide an overview of the hardware configuration and deployment cost, Table 4 summarizes the principal components used in the VISW Station and their approximate acquisition costs.

With the exception of the rain gauge and meteorological shield assembly, which were acquired as commercially available integrated products, the remaining hardware subsystems were assembled from individually sourced commercially available components and integrated into the monitoring platform developed for this study.

### 2.3. Data Acquisition Strategy

The embedded software responsible for environmental data acquisition was developed in Python and executed locally on the Raspberry Pi Zero 2 W platform running Raspberry Pi OS Bookworm Linux. The proposed architecture employed four main acquisition scripts: (i) an image acquisition script, (ii) a climatic monitoring script, (iii) a rainfall interrupt monitoring script, and (iv) an environmental acoustic recording script.

Air temperature and relative humidity measurements were obtained from the DHT22 sensor using the Adafruit_DHT library, while soil temperature measurements were acquired through the DS18B20 sensor using the 1-Wire communication interface available in the Linux operating system. Climatic measurements were sampled every two minutes and automatically stored as timestamped records in CSV files for subsequent environmental analysis. The DHT22 climatic sensor was connected to Raspberry Pi GPIO 6 (physical pin 31), while the DS18B20 soil temperature sensor was connected to GPIO 5 (physical pin 29).

Rainfall measurements obtained from the tipping-bucket rain gauge were acquired through GPIO 26 (physical pin 37) using digital interrupt detection. Each tipping event generated a hardware interrupt that incremented the rainfall count stored in a CSV file together with the corresponding date and time information. The rainfall monitoring script was automatically initialized during system boot and remained continuously active awaiting interrupt events throughout the monitoring operation.

The image acquisition system employed a modified OV5647 camera module with a mechanically controlled infrared filter. The filter actuation mechanism was controlled using an L298N H-bridge module connected to GPIO 8 (physical pin 24) and GPIO 7 (physical pin 26). During idle operation, both control pins were maintained at logical low level. To acquire RGB images, GPIO 8 was temporarily set to logical high level, supplying +3.3 V to position the infrared filter appropriately. After RGB image acquisition, both pins were briefly returned to logical low level before GPIO 7 was activated to apply reverse polarity and switch the camera into NOIR acquisition mode for Near-Infrared (NIR) image capture.

Acquisition routines were scheduled using the Linux Crontab service available in the Raspberry Pi OS Bookworm operating system. Images were acquired hourly between 06:00 and 18:00, always generating paired RGB and NOIR images for subsequent vegetation analysis. Environmental acoustic recordings were also scheduled through Crontab and executed every 10 min. Each audio recording had a duration of approximately 9 min and 55 s, allowing sufficient time for file closing procedures and initialization of subsequent recordings while minimizing resource conflict risks. The climatic acquisition script was configured to execute every two minutes, while the rainfall monitoring script operated continuously in background mode throughout the entire monitoring campaign.

All acquired data were stored locally using onboard storage devices due to the absence of communication infrastructure in several monitored areas. Temporal alignment between sensing modalities was achieved using timestamps generated by the Raspberry Pi system clock, enabling direct correlation between climatic variables, rainfall events, acoustic activity, and image-based vegetation dynamics throughout the monitoring period. The VISW stations did not include a dedicated hardware Real-Time Clock (RTC) module. Timekeeping was maintained using the Raspberry Pi system clock, while the operating system preserved the last known timestamp between reboots. Under normal operation, stations typically remained continuously powered for extended periods between maintenance visits, usually ranging from three to four months. The Raspberry Pi system clock was sufficient to maintain temporal consistency during these intervals, and no significant timestamp deviations were observed during normal operation. Timestamp inconsistencies were observed only in a small number of cases associated with prolonged power interruptions, and these events were identified during data quality control procedures through inconsistencies between recorded timestamps and environmental conditions observed in the acquired images and acoustic data. Future deployments will incorporate a dedicated RTC module to further improve temporal robustness during extended offline operation.

Considering the daily acquisition of 26 images with an average size of approximately 5 MB each and 144 compressed audio files with approximately 5 MB each, each VISW Station generates approximately 850 MB of data per day. After reserving approximately 4 GB for the operating system and auxiliary files, different microSD card capacities provide long-term autonomous storage operation ranging from approximately 2 to 9 months, depending on the selected storage configuration, as summarized in Table 5.

The stations operated entirely in offline mode, ensuring reliable operation even in remote environments without internet or cellular network availability.

Field maintenance and data retrieval procedures were performed through wireless local access using Secure Shell (SSH) connections. All monitoring stations were configured with a predefined Wi-Fi network name and password, allowing maintenance access through temporary hotspots generated using notebook computers or mobile devices during field visits. Once connected to the local wireless network, remote access to the Raspberry Pi Zero 2 W platform was established using the PuTTY SSH client, enabling direct access to acquisition scripts, Crontab scheduling routines, and system configuration parameters through Linux command-line interfaces.

File transfer operations could be performed remotely using the WinSCP software. However, due to the large amount of generated environmental data, remote wireless transfer was considered impractical for complete dataset retrieval under field conditions. A typical deployment period of approximately four months generated nearly 90 GB of environmental data per station, while the average transfer rate obtained through hotspot connections remained close to 2 MB/s, resulting in transfer times exceeding six hours for complete downloads.

For this reason, the adopted field maintenance strategy primarily consisted of physically removing the microSD card from the monitoring station and directly accessing the stored files using notebook computers running Linux operating systems. This approach enabled file transfer rates close to the native microSD read speed, typically around 70 MB/s depending on the storage device model, substantially reducing maintenance time during field operations.

After backup procedures and removal of previously collected datasets, the microSD card was reinserted into the monitoring station and the system clock was manually adjusted using Linux date synchronization commands before system reboot. Following initialization, timestamp generation was maintained locally using the Raspberry Pi system clock, enabling autonomous offline operation during the typical three- to four-month intervals between maintenance visits.

### 2.4. Acoustic Monitoring

Passive Acoustic Monitoring has emerged as a non-invasive and highly effective methodology for tracking biodiversity dynamics, particularly within complex tropical ecosystems undergoing ecological restoration. Low-cost autonomous acoustic monitoring devices, such as AudioMoth, have significantly expanded the accessibility of passive acoustic monitoring approaches for biodiversity and environmental assessment applications [22]. By capturing continuous audio data, PAM platforms record distinct biophonic and anthropogenic acoustic components that reflect habitat structure and species composition over extended temporal scales.

Deploying long-term acoustic monitoring hardware under remote field conditions presents substantial technical challenges, particularly regarding data storage, power limitations, and environmental sealing [23].

The integration of a digital Microelectromechanical Systems (MEMS) microphone directly interfaced through I2S protocol with an edge-enabled computing platform enabled low-power continuous acoustic acquisition under long-term remote deployment conditions.

Spectrograms were generated using Short-Time Fourier Transform (STFT) analysis [24,25] to represent temporal and spectral environmental soundscape dynamics.

False-color spectrograms were generated using the Ecoacoustics Audio Analysis software developed by Towsey and collaborators at the Queensland University of Technology (QUT) [26,27]. The spectrogram generation workflow consisted of the calculation of multiple ecoacoustic indices, including the Acoustic Complexity Index (ACI), associated with temporal variations in acoustic activity, Entropy (ENT), related to spectral and temporal signal distribution, and acoustic Events (EVN), representing discrete acoustic occurrences. These indices were subsequently mapped into RGB color channels. In the adopted configuration, ACI was assigned to the red channel, ENT to the green channel, and EVN to the blue channel, enabling visual discrimination of distinct temporal and spectral soundscape patterns.

Detailed processing parameters used for spectrogram generation are provided in Table A1 to ensure methodological reproducibility.

The acoustic analysis employed a custom parameter configuration, including a 24 kHz resampling rate, 512-sample frame length, linear frequency scaling, and frequency bands between 1 and 11 kHz. Audio recordings were segmented into 60 s intervals for standardized spectral analysis and acoustic index calculation.

Environmental acoustic recordings were stored in the Ogg Vorbis (OGG) compressed format to reduce storage requirements during long-term monitoring campaigns. Each 10 min recording generated an audio file of approximately 4.8 MB, resulting in 144 audio segments per day and enabling uninterrupted 24-h acoustic monitoring.

**Figure 6:**
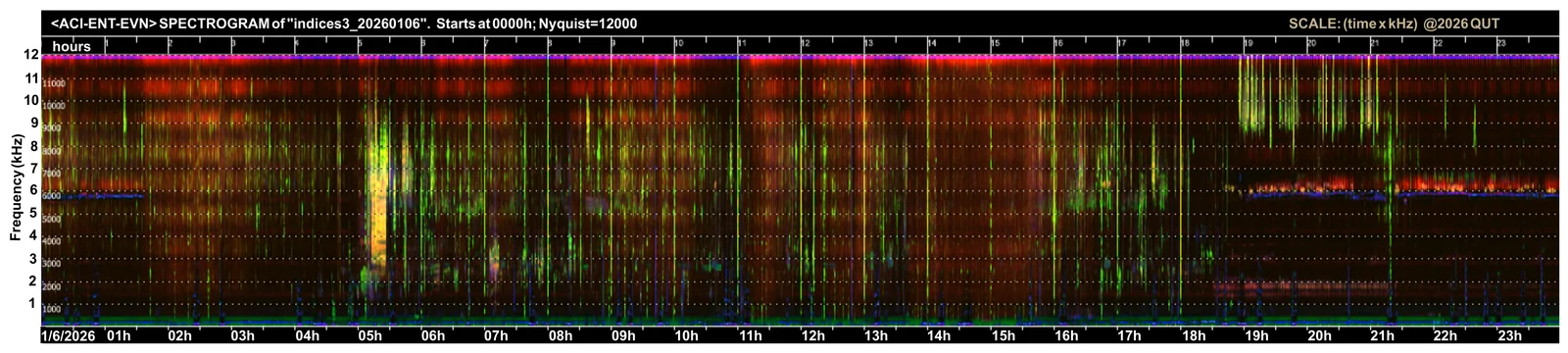
Example of an environmental spectrogram obtained from the passive acoustic monitoring on 6 January 2026, highlighting biological and environmental acoustic signatures. Ecoacoustic indices are mapped to RGB channels: ACI to red, ENT to green, and EVN to blue.

To investigate long-term acoustic dynamics, false-color spectrograms were generated from continuous audio recordings acquired by the VISW Station, as illustrated in Figure 6. Each spectrogram represents a complete 24 h monitoring period and was produced from 144 consecutive audio files with a duration of 10 min each, processed using the Analysis Program software. The resulting visualizations enabled the analysis of temporal and spectral patterns in the environmental soundscape over a full day of continuous monitoring. Different combinations of ecoacoustic indices generated distinct color patterns, highlighting changes in acoustic structure and soundscape composition throughout the analyzed recordings.

### 2.5. Vegetation Indices and Image Processing

Vegetation highlighting was achieved using RGB and NOIR images acquired by the proposed monitoring stations. Multiple vegetation indices were evaluated in order to investigate the potential of low-cost multispectral sensing for environmental monitoring applications.

In addition to microclimatic and acoustic dimensions, near-surface remote sensing provides critical high-resolution phenological data necessary to evaluate vegetation cover returns in degraded zones. While industrial multispectral cameras offer high precision, their prohibitive costs severely restrict dense spatial deployment in large-scale monitoring networks.

Recent developments demonstrate that low-cost smart cameras and modified Complementary Metal-Oxide-Semiconductor (CMOS) sensors, when integrated with software-controlled IR-CUT filters, represent a robust alternative for tracking multi-species tropical phenology and calculating accurate color-based vegetation indices [28]. This dual-band imaging approach allows the automated, sequential capture of visible (RGB) and near-infrared-sensitive frames under consistent lighting conditions without manual hardware adjustments.

The estimated infrared component was obtained by subtracting the red channel of the RGB image from the red channel of the NOIR image. Since the NOIR camera captures both visible red and near-infrared radiation, this procedure provides an approximate estimation of the infrared reflectance component:

(1) $$IR_{estimated}\approx R_{NOIR}-R_{RGB},$$

where $R_{NOIR}$ is the red-channel intensity extracted from the NOIR image, and $R_{RGB}$ is the red-channel intensity extracted from the corresponding RGB image.

It is important to emphasize that the proposed infrared estimation approach does not provide a physically calibrated near-infrared reflectance measurement. The approximation based on the subtraction between NoIR and RGB sensor responses represents a simplified empirical strategy intended to enhance relative infrared-sensitive information using low-cost commercial CMOS cameras. Due to the broad and partially overlapping spectral sensitivities of RGB and NoIR sensors, the estimated infrared component may still contain contributions from visible wavelengths and sensor-specific spectral responses. Therefore, the proposed method should be interpreted as a qualitative vegetation-sensitive proxy rather than a radiometrically calibrated multispectral measurement.

The normalized Excess Green Index (ExG), originally proposed by Woebbecke et al. [29], was calculated using the normalized RGB channels in order to enhance vegetation regions based on the relative contribution of the green channel:

(2) ExG=2g−r−b,

where *r*, *g*, and *b* denote the normalized red, green, and blue channel values, respectively.

Additionally, the Visible Atmospherically Resistant Index (VARI), proposed by Gitelson et al. [30], was evaluated as an alternative RGB-based vegetation enhancement approach due to its reduced sensitivity to illumination variations under visible-spectrum image acquisition conditions:

(3) $$VARI=\frac{G-R}{G+R-B},$$

where *R*, *G*, and *B* denote the red, green, and blue channel values, respectively.

**Figure 7:**
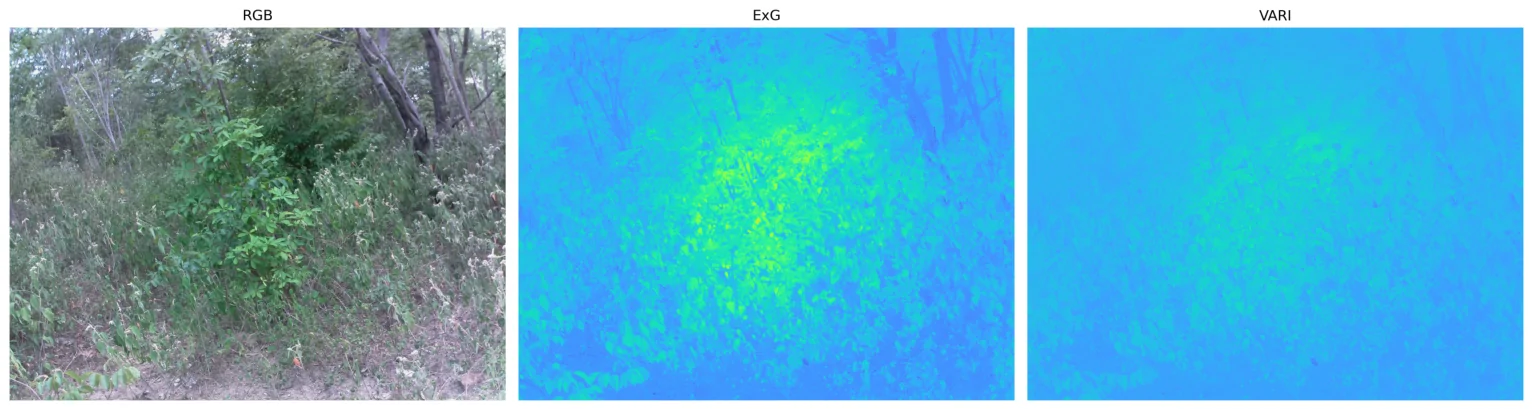
Comparison between the original RGB image and vegetation enhancement maps generated using the ExG and the VARI.

To comparatively evaluate the behavior of ExG and VARI under the environmental conditions investigated in this study, both indices were applied to representative RGB images acquired by the monitoring stations, as presented in Figure 7.

The two vegetation indices successfully highlighted vegetated areas under the environmental conditions evaluated in this study, as illustrated in Figure 7. ExG demonstrated superior contrast and more effective discrimination between vegetation and background elements and was consequently selected as the primary vegetation enhancement method for the long-term monitoring and temporal analysis presented throughout this manuscript.

A Hybrid Vegetation Index (HybridVI) was proposed by combining the normalized green component with the estimated infrared component:

(4) $$HybridVI=g+IR_{norm},$$

where *g* represents the normalized green-channel value and $IR_{norm}$ denotes the normalized estimated infrared component obtained from the difference between the red-channel responses of the NOIR and RGB images.

Additionally, a modified hybrid version of the ExG index, named HybridExG, was evaluated by incorporating the normalized infrared component as an additive reinforcement to the ExG formulation:

(5) $$HybridExG=ExG+0.5\times IR_{norm},$$

where ExG is the Excess Green Index, $IR_{norm}$ is the normalized estimated infrared component, and 0.5 is an empirical weighting coefficient adopted to balance the contribution of infrared information in the hybrid vegetation representation.

**Figure 8:**
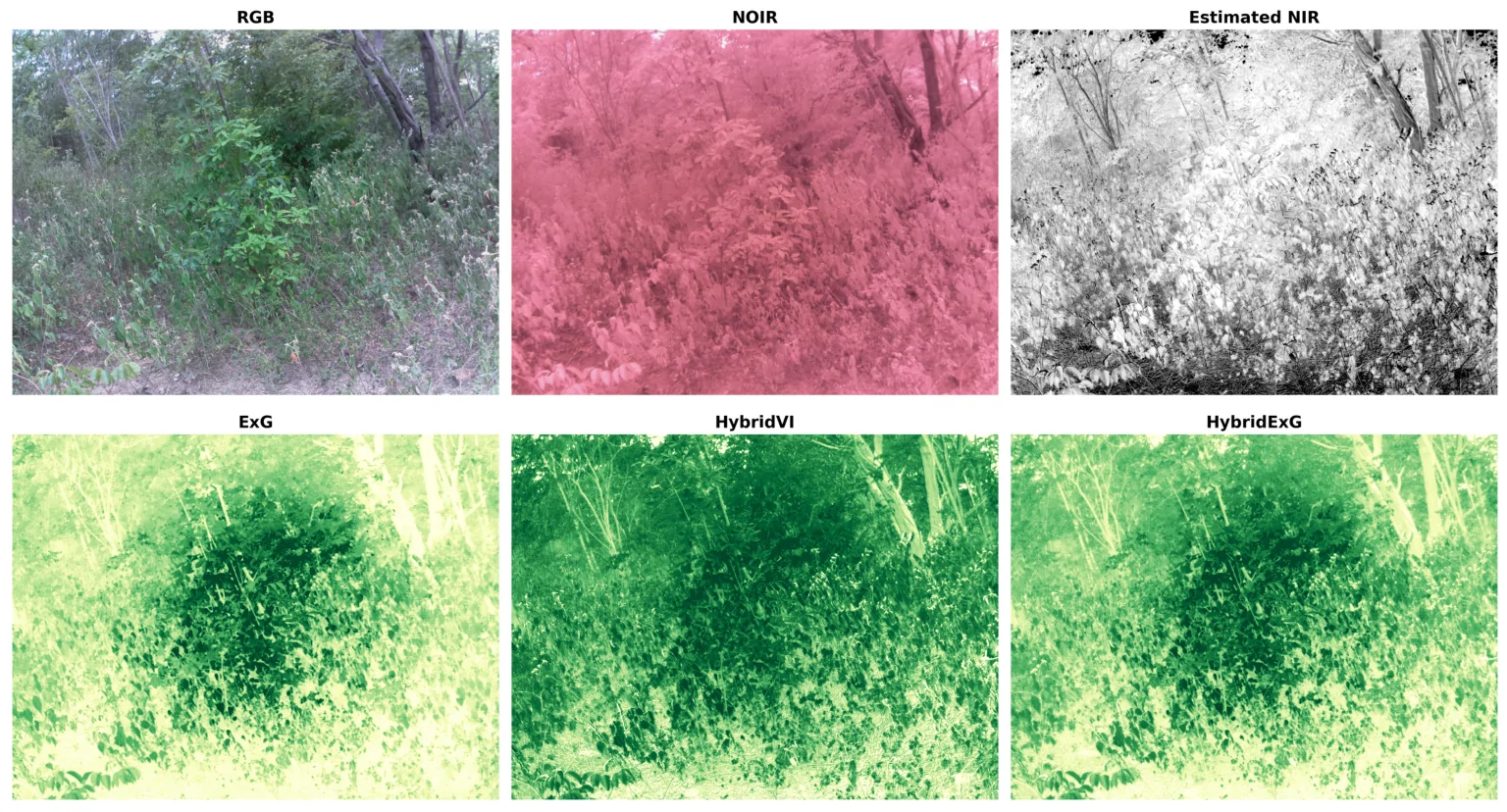
Comparison of RGB, NoIR, estimated infrared, ExG, HybridVI, and HybridExG vegetation representations derived from images acquired by the proposed VISW Station.

A qualitative comparison between RGB, NOIR, estimated infrared, ExG, HybridVI, and HybridExG vegetation representations obtained from images captured by the proposed VISW Station is presented in Figure 8.

The evaluated indices were compared in terms of vegetation enhancement capability and qualitative discrimination of vegetation structures under field conditions using low-cost RGB/NOIR camera integrated into the environmental monitoring station.

For temporal consistency, all images used in the ExG-based vegetation analysis were spatially aligned through image rotation correction, and a fixed Region Of Interest (ROI) was applied to all monthly comparisons in order to minimize variations caused by camera positioning and scene composition. Images acquired close to the 8th day of each month were selected for the analysis, preferentially using recordings obtained around 13:00 due to the more stable solar illumination conditions, reduced shadow influence, and higher incident solar radiation during this period.

The adopted preprocessing strategy was not intended to achieve perfect image registration or pixel-level alignment, but rather to provide a more consistent visual comparison between different periods of the year, emphasizing seasonal variations in vegetation conditions and landscape dynamics throughout the monitoring period.

## 3. Results

### 3.1. Long-Term Rainfall Dynamics

**Figure 9:**
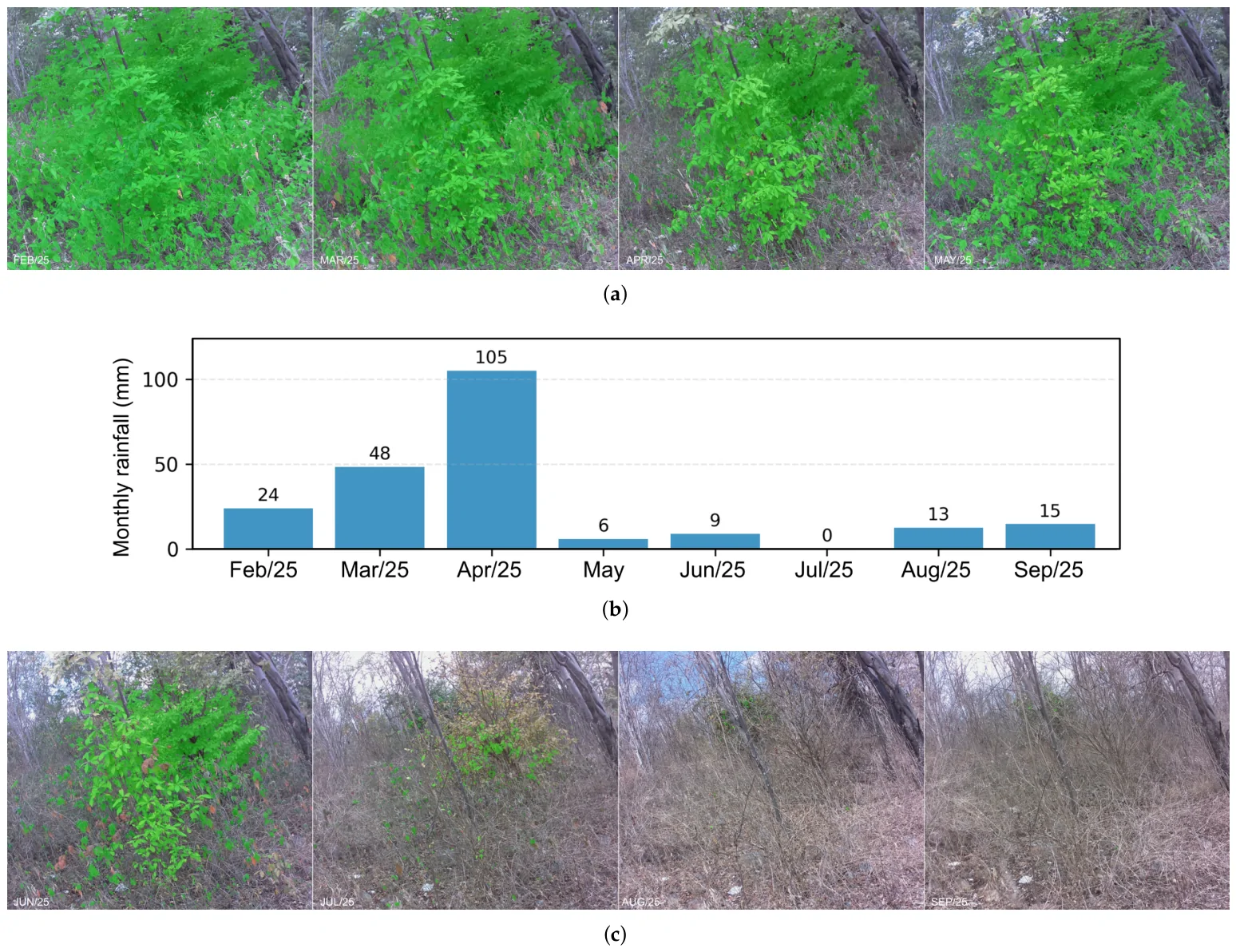
Temporal comparison between monthly ExG vegetation representations and accumulated rainfall between February 2025 and September 2025, highlighting seasonal vegetation dynamics under varying precipitation conditions. (**a**) Vegetation conditions observed between February and May 2025; (**b**) Monthly accumulated rainfall during the monitoring period; and (**c**) Progressive decline in vegetation density during the dry season.

The images presented in Figure 9 illustrate the temporal evolution of vegetation dynamics between February and September 2025 using environmental images acquired by the VISW Station at the Conselheiro Pena deployment location. As shown in Figure 9a, vegetation conditions between February and May 2025 remained strongly influenced by wet-season precipitation patterns. Figure 9b presents the accumulated rainfall throughout the monitoring period, while Figure 9c highlights the progressive reduction in vegetation density observed between June and September 2025 during the transition toward dry seasonal conditions. Although a reduction in vegetation density would normally be expected in May due to the progressive transition toward the regional dry season, the elevated rainfall observed during April resulted in increased vegetation development and greener ExG responses during May. After this period, vegetation density progressively decreased as precipitation levels declined, with vegetation signatures becoming substantially reduced by September under prolonged dry conditions.

All images shown in Figure 9 were acquired by the VISW Station at the Conselheiro Pena deployment location. Vegetation enhancement representations were subsequently generated through offline image processing.

**Figure 10:**
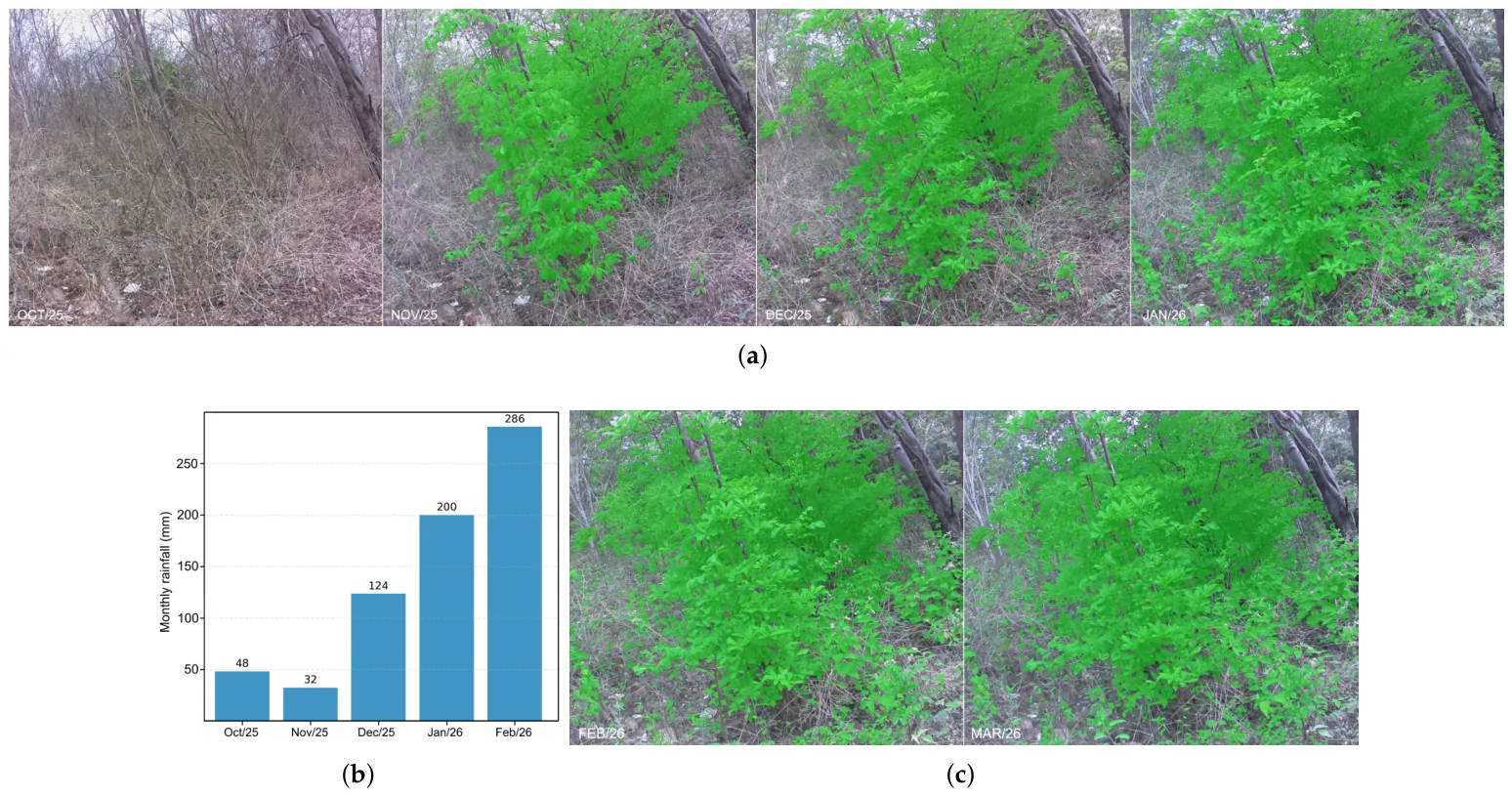
Temporal evolution of ExG-based vegetation representations between October 2025 and March 2026 at the Conselheiro Pena deployment location, illustrating the progressive recovery of vegetation density associated with the return of rainfall during the wet season. (**a**) Initial vegetation recovery stages observed between October 2025 and January 2026; (**b**) monthly accumulated rainfall during the monitoring period; and (**c**) intensified vegetation development observed between February and March 2026 under increased precipitation conditions.

Vegetation density continued to increase between October and November 2025 despite the reduction in monthly precipitation, as illustrated in Figure 10. This behavior is consistent with the delayed response of vegetation to accumulated rainfall observed in the correlation analysis.

This behavior suggests that the rainfall accumulated in October was sufficient to increase visible vegetation cover, allowing continued plant development and greening responses even under reduced precipitation conditions. Such delayed vegetation responses are commonly associated with residual soil moisture availability and progressive physiological recovery of plant communities after prolonged dry periods.

### 3.2. Green View Index (GVI) Estimation and Rainfall Relationship

To demonstrate the potential of the VISW Station for long-term vegetation monitoring, a preliminary analysis was conducted using the GVI. The GVI is a widely adopted metric that quantifies the proportion of visible vegetation within an image and has been used to assess landscape characteristics, vegetation abundance, and environmental quality from photographic observations [31,32].

The concept of evaluating visible greenery from image-based observations was initially introduced by Aoki [31], who proposed quantitative methods for assessing landscape greenery. Subsequently, Yang et al. [32] formalized the GVI as the proportion of visible green vegetation within an image, demonstrating its applicability for vegetation assessment using digital photography. Derived directly from visual observations, the GVI provides a practical and computationally efficient indicator for continuous environmental monitoring using low-cost camera systems.

In this study, the GVI was estimated from RGB images acquired by the VISW Station at the Conselheiro Pena monitoring site. The analysis utilized the same images previously processed for vegetation enhancement, which had already undergone geometric alignment and ROI extraction. Vegetation pixels were identified using an empirical RGB thresholding procedure. Pixels were classified as vegetation when satisfying the conditions (G > 140), (R < 180), (B < 120), and ((G-R) > 20), where (R), (G), and (B) correspond to the red, green, and blue channel intensities, respectively.

The GVI was calculated as the percentage of pixels classified as vegetation within the selected ROI:

(6) $$GVI=\frac{N_{veg}}{N_{total}}\times 100,$$

where $N_{veg}$ is the number of pixels classified as vegetation and $N_{total}$ is the total number of pixels within the ROI.

Table 6 summarizes the monthly rainfall measurements and corresponding GVI values obtained throughout the monitoring period.

**Figure 11:**
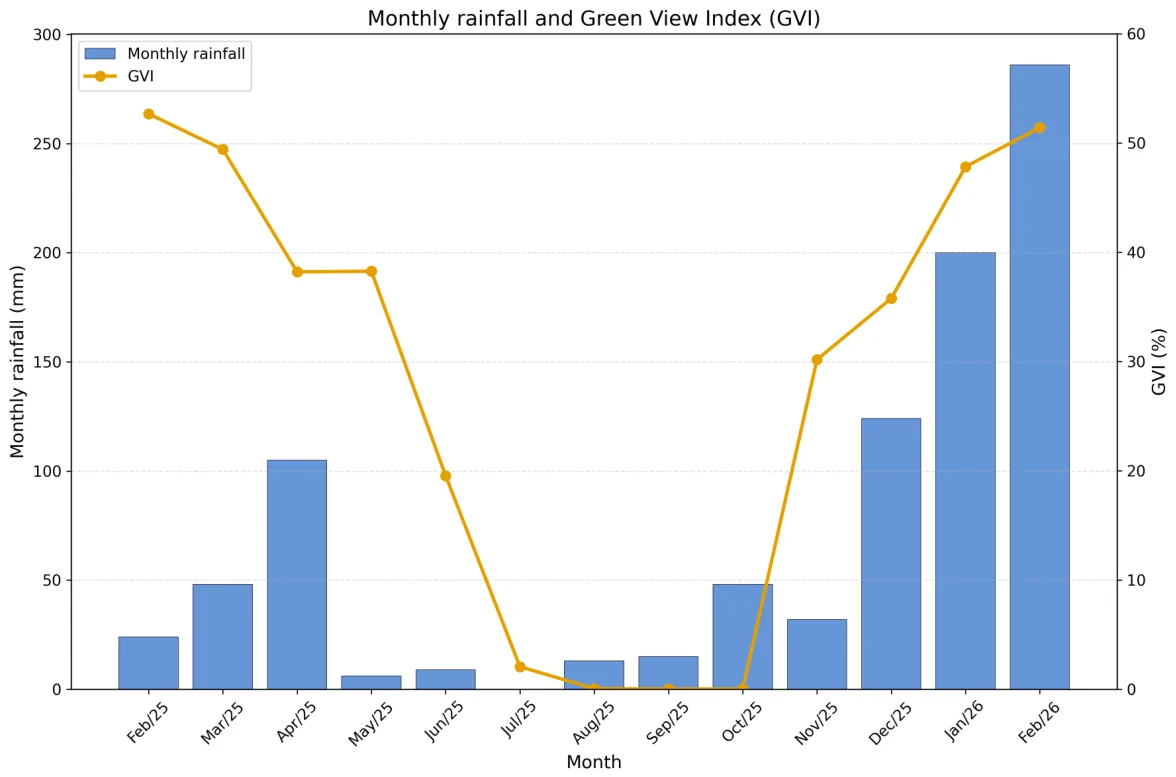
Monthly rainfall and Green View Index (GVI) values measured at the Conselheiro Pena monitoring station between February 2025 and February 2026.

Figure 11 presents the temporal evolution of monthly rainfall and GVI values. A pronounced seasonal pattern can be observed. Vegetation greenness progressively decreased during the dry season, reaching minimum values between August and October 2025, and subsequently recovered following the return of more frequent rainfall events.

To further investigate the relationship between rainfall and vegetation greenness, correlation analyses were performed considering multiple antecedent precipitation windows. Pearson’s correlation coefficient and Spearman’s rank correlation coefficient were calculated using same-month rainfall as well as cumulative rainfall from previous months.

The strongest relationship was observed when considering the cumulative rainfall of the two preceding months, yielding Pearson’s (r = 0.80) and Spearman’s ρ=0.88.

The statistical analysis indicated that antecedent rainfall explained vegetation greenness more effectively than precipitation occurring during the same month. The highest correlation coefficients were obtained for the cumulative rainfall of the two preceding months, suggesting that vegetation response was more strongly associated with short-term accumulated water availability than with immediate precipitation events. Although based on a limited dataset from a single monitoring location, these results demonstrate the ability of the VISW Station to continuously acquire environmental data and generate quantitative indicators suitable for supporting long-term vegetation monitoring and future ecosystem recovery studies in post-disaster environments.

### 3.3. Climatic Variability

**Figure 12:**
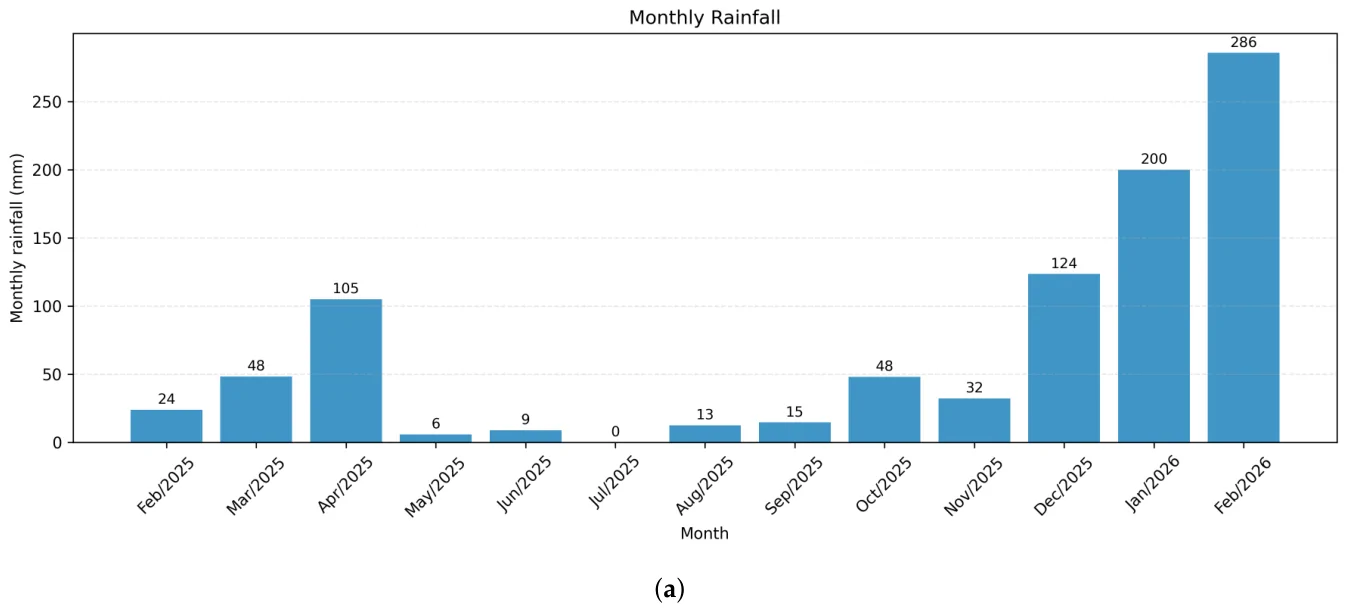

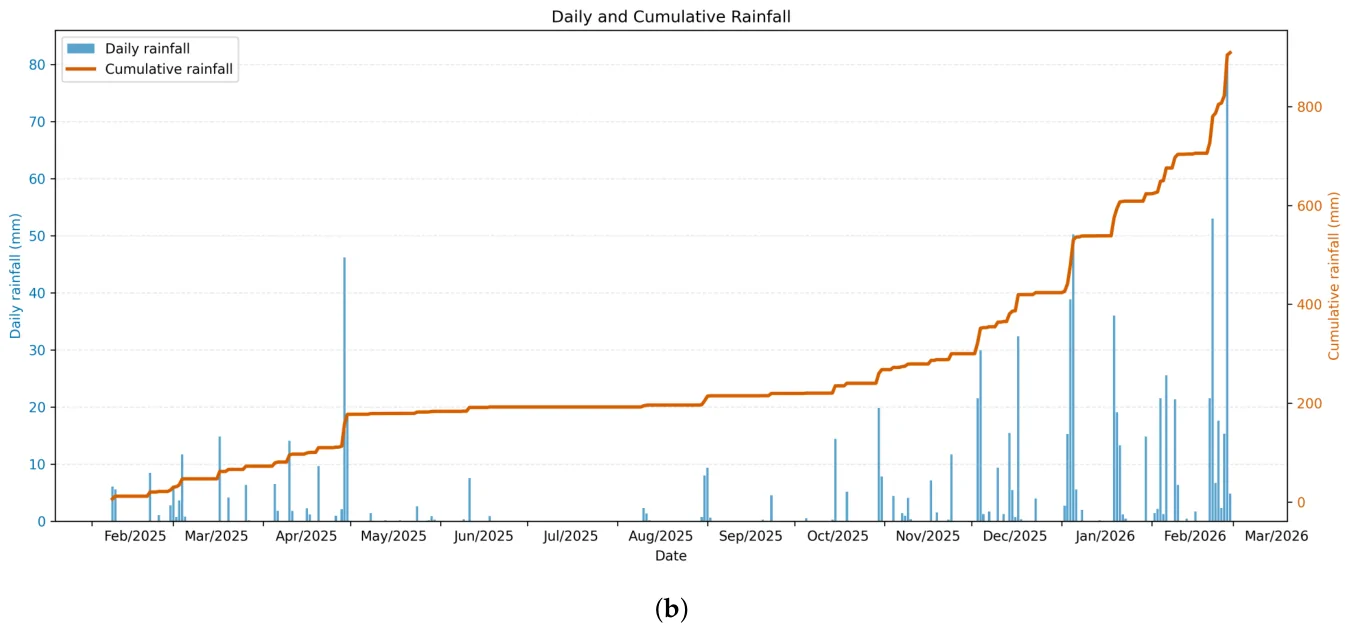
Temporal rainfall dynamics recorded at the Conselheiro Pena monitoring station between February 2025 and March 2026: (**a**) monthly accumulated precipitation and (**b**) daily rainfall measurements together with the cumulative precipitation throughout the monitoring period.

The climatic analysis demonstrated clear daily and seasonal variations in environmental conditions. Figure 12 presents the temporal dynamics of precipitation between February 2025 and March 2026, including (a) monthly accumulated precipitation and (b) daily rainfall measurements together with the cumulative precipitation throughout the monitoring period. The obtained results demonstrate a strong seasonal rainfall pattern, characterized by elevated precipitation during the wet season and a pronounced reduction in rainfall during the dry months.

Daily rainfall measurements revealed the occurrence of short-duration high-intensity precipitation events concentrated mainly between October 2025 and March 2026, while monthly accumulated rainfall highlighted the progressive transition between dry and wet seasonal conditions. The cumulative rainfall curve further illustrates the temporal distribution of precipitation throughout the monitoring campaign, allowing direct comparison with vegetation dynamics and environmental acoustic activity observed in the monitored ecosystems.

The prolonged reduction in precipitation observed between May and September 2025 was associated with substantial decreases in vegetation density and ExG responses, while the return of rainfall after October 2025 promoted progressive vegetation recovery and increased biological activity across the monitored areas.

**Figure 13:**
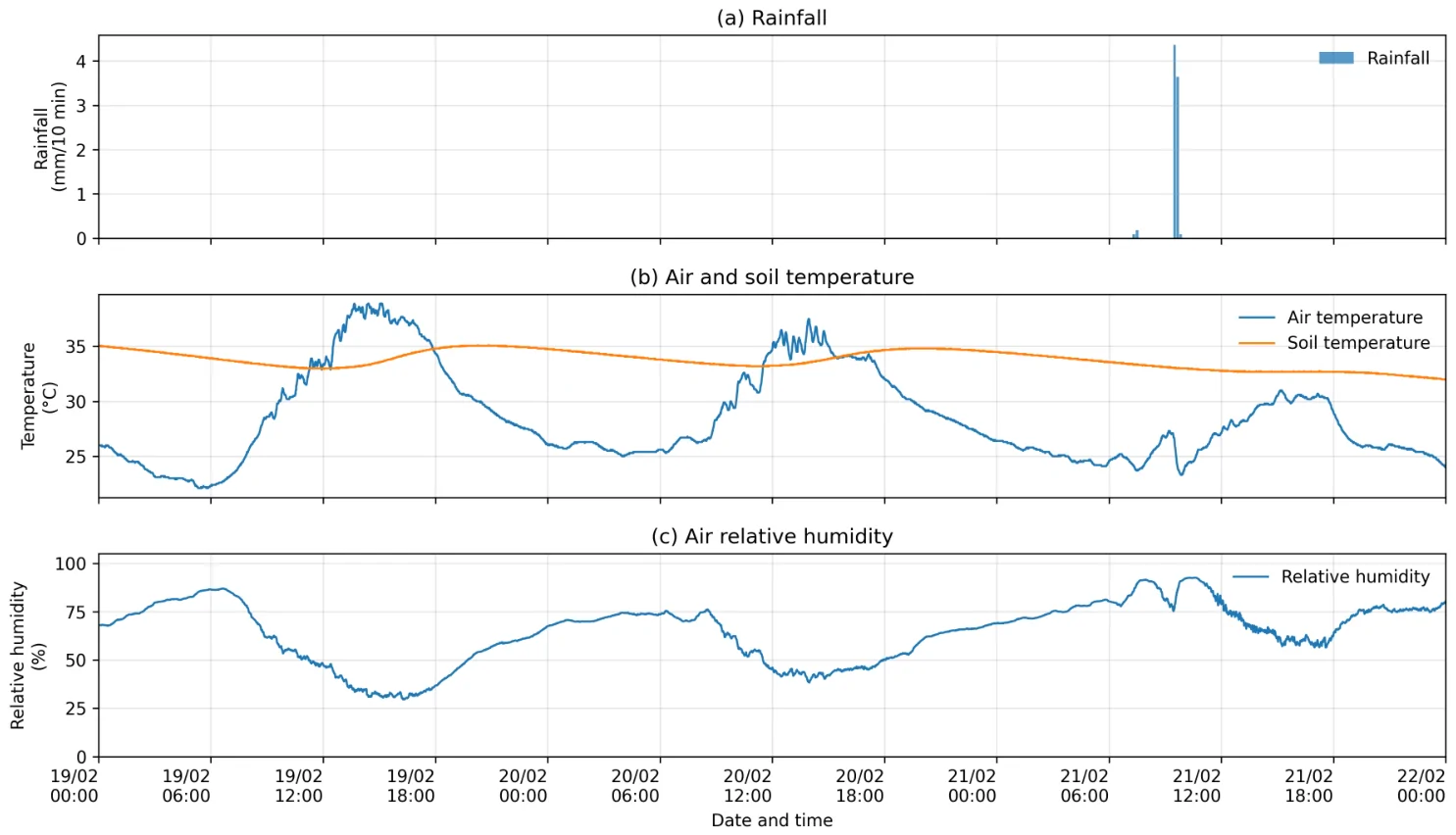
Example of short-term climatic dynamics recorded by the Conselheiro Pena environmental monitoring station between 19 and 22 February 2025: (**a**) rainfall intensity, (**b**) air and soil temperature variations, and (**c**) air relative humidity measurements.

The short-term climatic dynamics recorded by the VISW station between 19 and 22 February 2025 are illustrated in Figure 13. The obtained results demonstrate the capability of the proposed system to continuously monitor rapid environmental variations under field conditions.

The rainfall events observed around midday on 21 February were associated with simultaneous reductions in air temperature and increases in relative humidity, demonstrating the expected interaction between precipitation and local microclimatic conditions. Air temperature exhibited pronounced daily cycles, with maximum values occurring during periods of higher solar incidence and minimum values during nighttime periods. In contrast, soil temperature presented smoother temporal variations due to the higher thermal inertia of the soil environment.

Relative humidity measurements showed an inverse relationship with air temperature throughout most of the monitoring period, with humidity levels increasing during rainfall events and nighttime conditions. These results demonstrate the potential of the proposed monitoring system for continuous environmental characterization and microclimatic analysis in remote ecosystems.

The short-term climatic dynamics monitored by the VISW station, including (a) rainfall intensity, (b) air and soil temperature variations, and (c) air relative humidity measurements (Figure 13), revealed a delayed thermal response of the soil environment. While air temperature rapidly increased under solar radiation, soil temperature exhibited slower variations due to soil thermal inertia. After decreases in air temperature, soil temperature continued increasing for a short period before gradually decreasing again, reflecting heat storage and delayed thermal dissipation processes within the soil.

**Figure 14:**
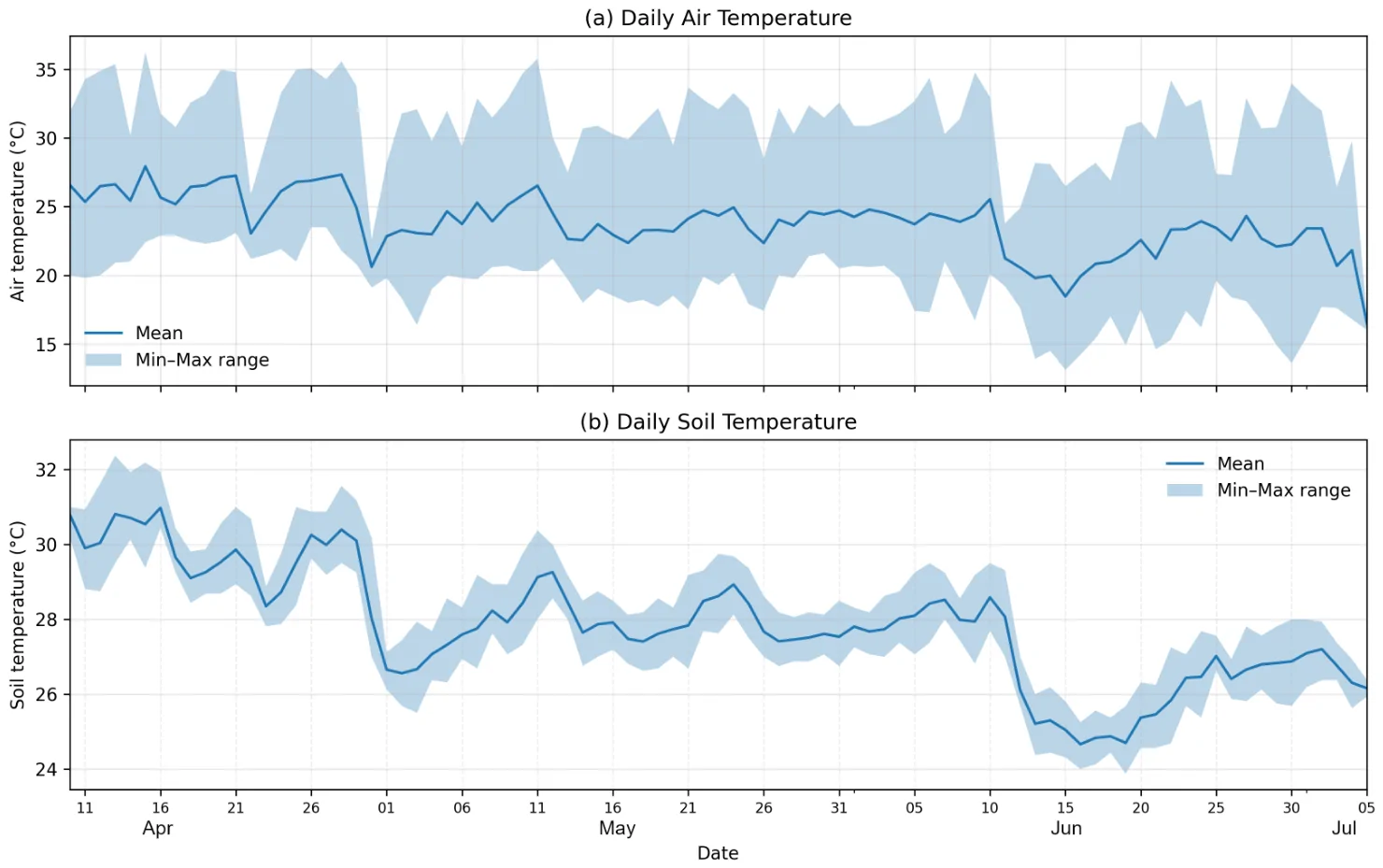
Temporal evolution of daily temperature dynamics recorded at the Conselheiro Pena environmental monitoring station between April and July 2025: (**a**) daily air temperature and (**b**) daily soil temperature.

Differences between atmospheric and soil thermal dynamics throughout the monitoring period, including (a) daily air temperature and (b) daily soil temperature, are presented in Figure 14. Solid lines represent daily mean values, while shaded regions indicate the minimum and maximum temperature ranges. Air temperature exhibited substantially larger daily thermal amplitudes, with pronounced differences between minimum and maximum values due to the rapid response of the atmosphere to solar radiation and short-term environmental variations. In contrast, soil temperature presented smoother variations and reduced thermal amplitude, reflecting the higher thermal inertia of the soil environment and its slower response to heating and cooling processes.

The obtained results also indicate that daily mean temperatures generally remained closer to minimum temperature values, particularly for air temperature measurements. This behavior is associated with the predominance of lower nighttime temperatures over longer periods, while daytime high-temperature events occurred as relatively short-duration peaks that were insufficient to significantly elevate the overall daily average. Such thermal behavior highlights the influence of diurnal heating cycles and soil heat storage processes on local microclimatic conditions.

### 3.4. Ecoacoustic Analysis

**Figure 15:**
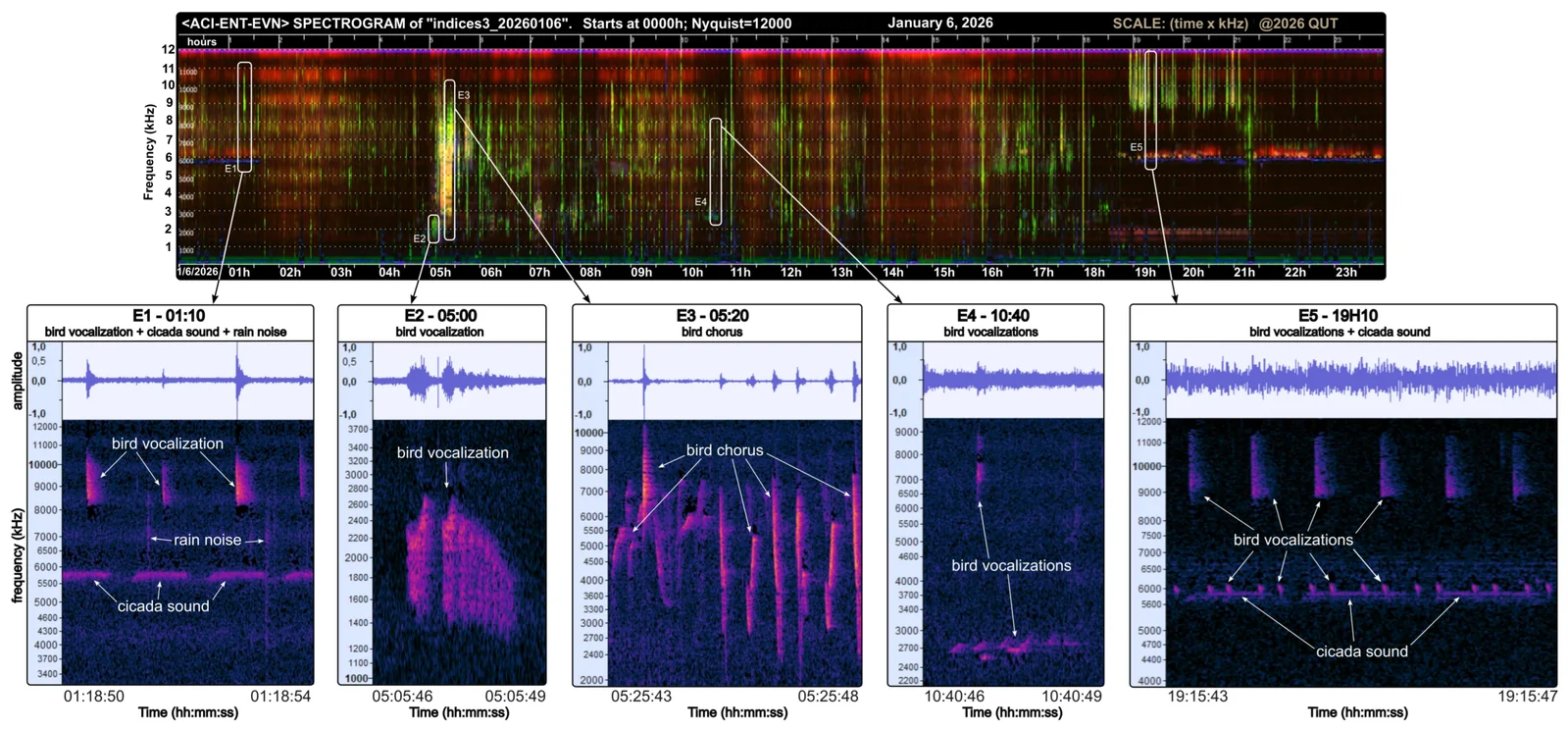
False-color environmental spectrogram generated from passive acoustic recordings acquired on 6 January 2026 at the Conselheiro Pena monitoring station. Ecoacoustic indices are mapped to RGB channels: ACI to red, ENT to green, and EVN to blue.

A daily false-color spectrogram generated from passive acoustic recordings obtained on 6 January 2026 is presented in Figure 15. The spectrogram allows the visualization of temporal and spectral variations in environmental soundscapes throughout the entire day, demonstrating the capability of the proposed monitoring system to continuously capture biological and environmental acoustic dynamics.

Distinct acoustic patterns associated with bird vocalizations, bird chorus activity, cicada sounds, and rainfall events can be identified at different periods of the day. Early morning periods exhibited intense bird chorus activity concentrated mainly between approximately 3 and 9 kHz, while cicada acoustic signatures were observed as persistent narrow-band frequency components around 5–6 kHz during warmer periods of the day.

Rainfall events generated broadband acoustic signatures distributed across multiple frequency bands, producing high-intensity spectral responses distinguishable from biological sounds. The detailed spectrogram excerpts (E1–E5) further illustrate representative acoustic events extracted from specific time intervals, highlighting the ability of the system to detect and differentiate distinct environmental acoustic sources under real field conditions.

The obtained results demonstrate the potential of long-term passive acoustic monitoring for environmental characterization, biodiversity assessment, and identification of temporal ecological patterns in remote ecosystems.

Temporal and spectral variations in biological and environmental acoustic activity were observed throughout the analyzed 24 h period, including representative examples of bird vocalizations, bird chorus activity, cicada sounds, and rainfall-related acoustic signatures extracted from specific time intervals (E1–E5), as illustrated in Figure 15. Event E5, recorded between approximately 19:10 and 19:20, was particularly representative of the crepuscular transition period, revealing the simultaneous occurrence of bird vocalizations and continuous cicada emissions. Detailed spectrogram inspection showed that cicada emissions generated relatively continuous and spectrally stable acoustic bands, whereas bird vocalizations appeared as short-duration broadband events with greater temporal variability.

Additionally, some bird vocalizations exhibited more harmonic and spectrally structured patterns, characterized by the presence of stable frequency components and harmonic organization over time. These characteristics contributed to increased spectral entropy responses and consequently generated greener regions in the false-color spectrogram representation.

These distinct temporal and spectral characteristics resulted in different ecoacoustic index combinations and consequently produced different color patterns in the false-color spectrogram representation. Although both acoustic sources were biologically related, their distinct sound structures generated different responses in the ACI, ENT, and EVN indices, reinforcing the capability of false-color ecoacoustic visualization techniques to discriminate different environmental acoustic behaviors.

**Figure 16:**
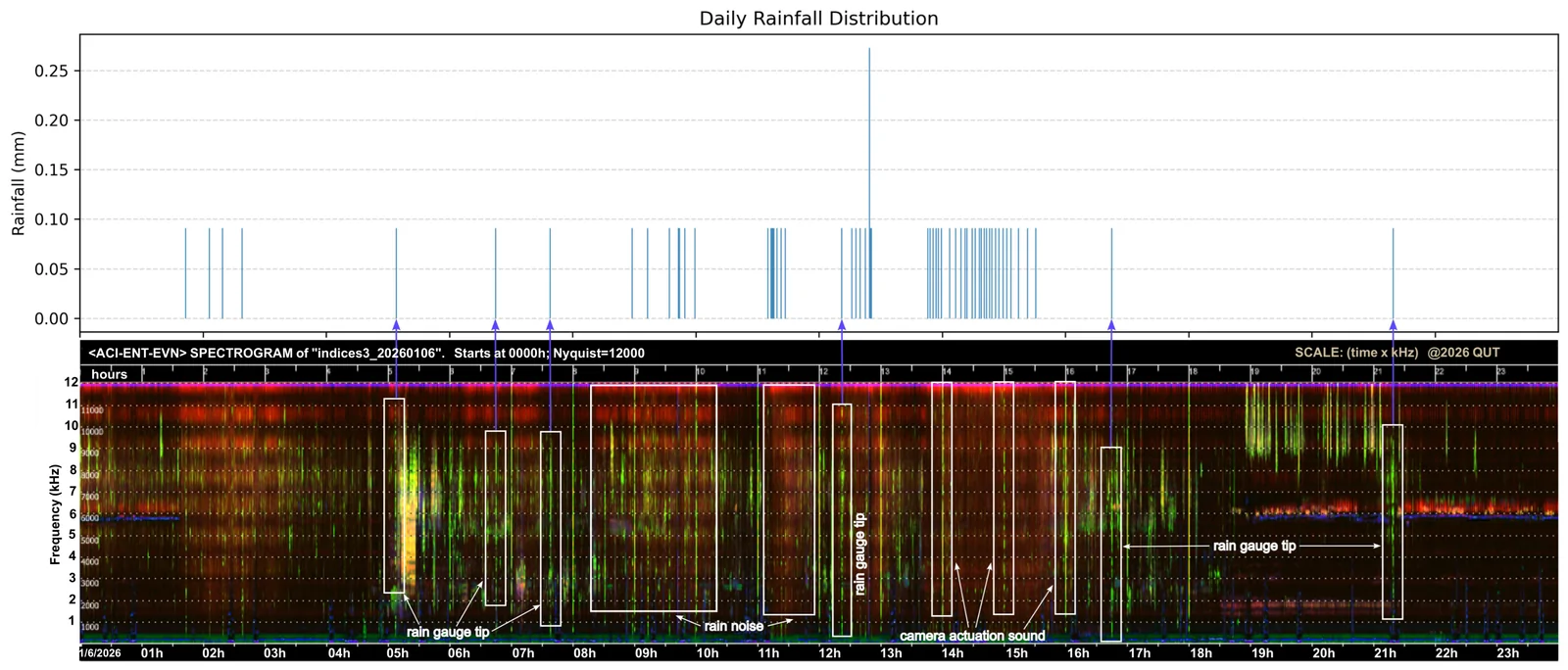
Comparison between rainfall measurements and passive acoustic recordings obtained on 6 January 2026, at the Conselheiro Pena monitoring station. Ecoacoustic indices are mapped to RGB channels: ACI to red, ENT to green, and EVN to blue.

The comparison between rainfall measurements and the corresponding environmental spectrogram generated from passive acoustic recordings is presented in Figure 16. The results demonstrate the capability of the proposed monitoring system to simultaneously capture climatic and acoustic events, enabling direct correlation between rainfall occurrence and environmental soundscape dynamics.

Distinct acoustic patterns associated with tipping-bucket rain gauge activations can be identified in the spectrogram as short-duration broadband structures occurring synchronously with precipitation events. Rainfall periods also produced continuous high-intensity spectral responses distributed across multiple frequency bands, represented mainly by red-colored regions in the false-color spectrogram.

In addition, periodic acoustic events generated by the mechanical actuation of the imaging system during hourly image acquisition procedures were consistently detected by the passive acoustic monitoring module. The identification of both environmental and system-generated acoustic patterns demonstrates the temporal synchronization capability of the proposed multimodal monitoring architecture and highlights the potential for integrated environmental event analysis using combined climatic and acoustic datasets.

Acoustic signatures associated with tipping-bucket rain gauge activations, rainfall noise, and periodic camera actuation sounds related to hourly image acquisition events were identified throughout the analyzed recordings, as illustrated in Figure 16. The rain gauge tipping mechanism generated impulsive broadband acoustic structures with energy distributed across multiple frequency bands. These events increased spectral entropy responses, producing greener patterns in the false-color spectrogram representation and facilitating the identification of rainfall occurrences within the continuous acoustic dataset.

### 3.5. Operational Challenges and System Robustness

**Figure 17:**
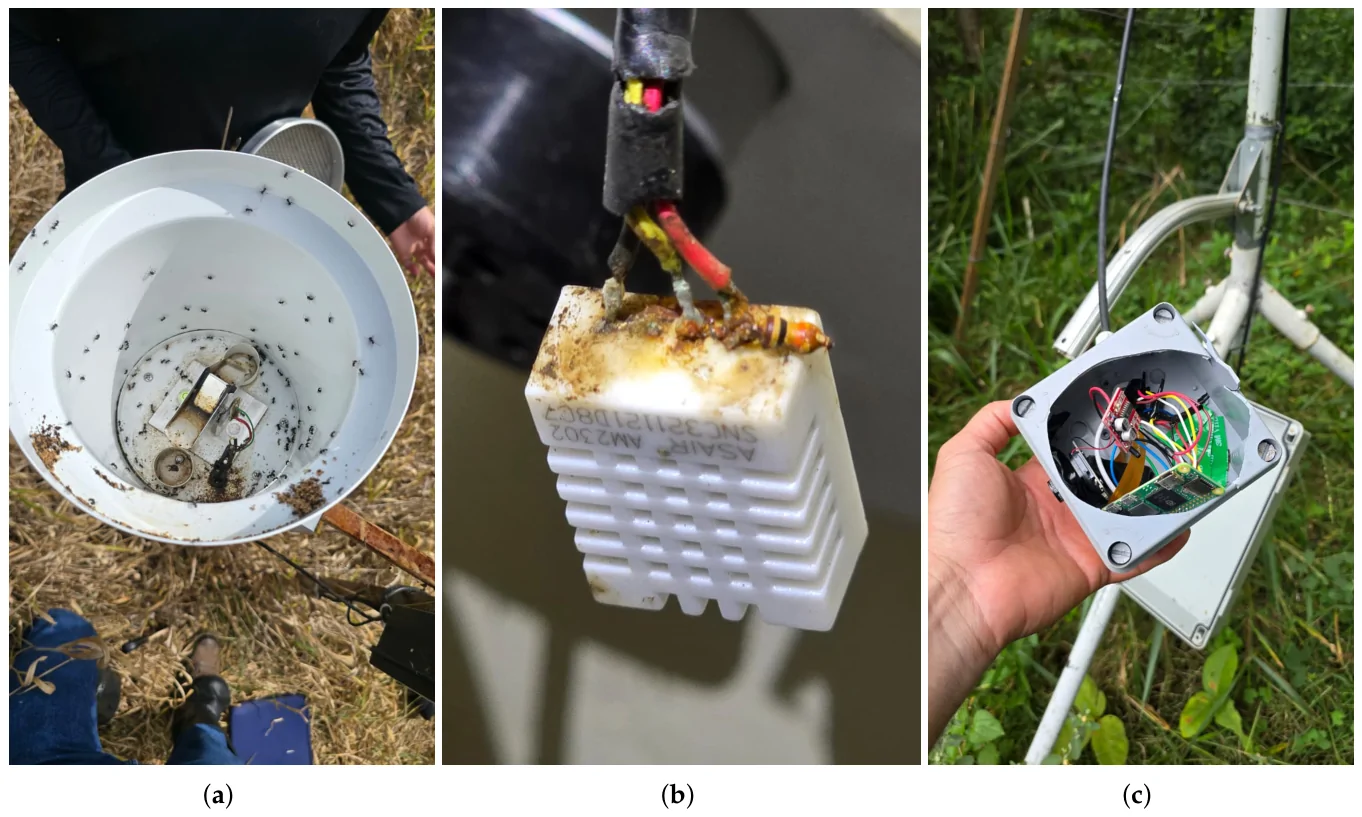
Examples of operational challenges observed during long-term field deployment of the environmental monitoring stations. (**a**) ant infestation affecting the tipping-bucket rain gauge mechanism, (**b**) electronic damage caused by ants, and (**c**) theft of one monitoring station during the field deployment period.

Throughout more than 18 months of continuous field deployment, only one monitoring station was lost due to theft (Figure 17c), demonstrating the relatively high operational resilience of the proposed system under real environmental conditions. Long-term deployment exposed the monitoring stations to multiple environmental challenges, including humidity, insects, vegetation growth, and wildlife interaction.

Additional operational issues observed during field deployment included ant infestation inside the tipping-bucket rain gauge, as illustrated in Figure 17a. The obstruction of the water drainage system, occasionally caused by the accumulation of leaves from nearby trees, created favorable conditions for ant infestation through the lower water outlet openings of the pluviometer structure. In some situations, these events partially affected the rain gauge operation, requiring preventive maintenance procedures to preserve measurement reliability and long-term data acquisition stability.

Another important operational challenge observed during long-term deployment was related to data storage reliability. Initial versions of the monitoring stations employed conventional microSD cards for continuous environmental data recording. However, after approximately six months of uninterrupted operation, several storage devices presented permanent failures caused by intensive write cycles associated with continuous acquisition of climatic, acoustic, and image datasets. In some cases, complete environmental datasets were partially or entirely lost due to storage corruption.

To improve long-term reliability, the conventional storage devices were replaced by High Endurance microSD cards specifically designed for continuous recording applications. These storage devices demonstrated substantially improved durability under continuous field operation and reduced the occurrence of storage-related failures throughout the monitoring campaign.

One representative example is presented in Figure 17b, where ant activity caused damage to the electronic circuitry associated with the DHT22 climatic sensor. In this case, ants damaged the 10 kΩ pull-up resistor required for sensor communication, temporarily interrupting temperature and humidity measurements. Such events highlight the importance of robust enclosure sealing, preventive maintenance procedures, and long-term field validation for autonomous environmental monitoring systems operating in remote ecosystems.

Several operational challenges were encountered during the long-term field deployment (Figure 17). These included (a) ant infestation affecting the tipping-bucket rain gauge mechanism, (b) electronic damage caused by ants, including destruction of the 10 kΩ pull-up resistor associated with the DHT22 climatic sensor, and (c) the theft of one monitoring station during the field deployment period. Despite these issues, the remaining stations continued operating and provided valuable long-term environmental datasets, highlighting both the practical challenges of autonomous monitoring in remote environments and the robustness of the proposed platform.

#### Long-Term Deployment Performance

The deployment schedule of the VISW monitoring network is summarized in Table 7. Stations were progressively installed between September 2024 and February 2025, allowing the evaluation of the proposed platform under different environmental and seasonal conditions. Consequently, the operational records obtained from individual stations ranged from approximately 12 to more than 18 months of field deployment.

The amount of data collected by each VISW station throughout the monitoring campaign is summarized in Table 8. Between September 2024 and February 2026, the monitoring network generated approximately 1.79 TB of multimodal environmental data, including acoustic recordings, climatic measurements, and RGB/NoIR imagery.

The long-term deployment provided valuable insights into the operational robustness of the platform. Despite the challenges inherent to continuous outdoor operation, the monitoring network remained active throughout the study period. The only permanent station loss resulted from a theft event at the bufalo site.

Particularly noteworthy was the performance observed during the final monitoring campaign (November 2025 to February 2026). Excluding the Bufalo station, which had previously been lost due to theft, all remaining stations successfully acquired environmental data throughout the collection period. Together, the eight active stations generated approximately 680.24 GB of multimodal data, representing the largest data volume obtained during a single campaign. This result demonstrates the operational maturity achieved by the VISW network after successive hardware refinements and maintenance interventions, highlighting its suitability for long-term environmental monitoring applications.

During maintenance visits, some stations were found with battery voltages below 9 V. As the battery packs consisted of three series-connected lithium-ion cells (3S configuration), voltages below this threshold indicated deep-discharge conditions potentially associated with permanent battery damage. In such cases, the affected battery pack was replaced with a spare unit. Stations installed in locations with adequate solar exposure generally operated throughout the monitoring campaign without apparent battery-related failures. Although battery capacity was not periodically measured and some degree of aging may have occurred, no systematic reduction in operational autonomy was observed under adequate solar irradiation conditions.

In most cases, energy-related interruptions were associated with gradual reductions in energy availability, eventually resulting in battery discharge and station shutdown. Under these conditions, stations typically remained inoperative until maintenance activities were performed and the battery pack was replaced. However, a small number of stations experienced intermittent restart events associated with unstable power conditions. In these situations, temporary loss of temporal synchronization occasionally occurred, causing inconsistencies between recorded timestamps and actual environmental conditions, such as daytime images being associated with nighttime timestamps. These events were identified during data quality-control procedures and corrected prior to analysis. The multimodal nature of the collected dataset, particularly the combination of image records and environmental acoustic patterns, facilitated the identification and verification of such temporal inconsistencies.

An additional challenge identified during long-term operation was the variability of solar exposure at stations deployed near vegetation. Throughout the monitoring period, two environmental factors were observed to alter the solar conditions initially considered during installation and maintenance activities. The first was the seasonal variation in solar geometry, particularly during the winter months (approximately April to August), which modified the duration and incidence angle of sunlight reaching the photovoltaic panels. The second was the rapid growth of surrounding vegetation during the rainy season (typically from December to February), which progressively reduced the size of natural clearings and increased shading from nearby branches and foliage.

These effects were considerably less pronounced in stations installed in open areas, where solar exposure remained relatively stable throughout the year. Together, these observations highlight the challenges associated with maintaining long-term photovoltaic energy generation in partially shaded environments. To improve energy resilience, the initial 10 W photovoltaic panels used in all stations were progressively replaced by 30 W panels in locations where energy availability proved insufficient. The larger panels substantially improved operational reliability and contributed to the successful operation of the monitoring network during the final monitoring campaigns.

Software reliability was primarily achieved through the use of independent acquisition scripts scheduled by the Linux Crontab service. Following power restoration or system reboot, all monitoring routines were automatically reinitialized, allowing image, climatic, rainfall, and acoustic acquisition to resume without manual intervention. The use of independent acquisition processes also reduced the impact of individual task failures on the overall monitoring operation.

Throughout the monitoring campaign, occasional maintenance interventions were required due to environmental exposure, sensor degradation, storage media failures, and power-related issues. Nevertheless, the increasing amount of data collected during successive campaigns reflects the progressive refinement of both the platform and maintenance procedures. Overall, the results demonstrate the feasibility of long-term multimodal environmental monitoring using low-cost hardware under real-world deployment conditions.

## 4. Discussion

The obtained results demonstrate the feasibility of using low-cost multimodal environmental monitoring systems for long-term environmental monitoring and ecosystem assessment in remote and post-disaster environments. Unlike conventional environmental monitoring approaches that rely on communication infrastructure and centralized processing, the proposed platform operated continuously in fully offline mode while simultaneously acquiring climatic, acoustic, and image-based environmental data.

The integration of multiple sensing modalities enabled the identification of relationships between precipitation dynamics, vegetation behavior, and environmental acoustic activity. The observed seasonal variations in vegetation greenness were associated with rainfall patterns recorded by the climatic sensors. Correlation analysis demonstrated that vegetation greenness responded more strongly to accumulated precipitation from preceding months than to rainfall recorded during the same month. The strongest relationship was observed when GVI values were compared with cumulative rainfall from the two preceding months (Pearson’s r=0.80; Spearman’s ρ=0.88), suggesting a delayed vegetation response to water availability. Periods of reduced precipitation coincided with progressive decreases in vegetation density and greenness, while the return of rainfall during the wet season promoted vegetation recovery. These findings reinforce the potential of low-cost image-based vegetation indices for long-term phenological monitoring under field conditions.

The acoustic monitoring results further demonstrated the capability of the proposed system to characterize environmental soundscapes and detect distinct ecological and climatic events. Bird vocalizations, cicada activity, rainfall events, and anthropogenic sounds produced clearly distinguishable spectral signatures in the generated false-color spectrograms. The ability to identify temporal patterns in biological acoustic activity highlights the potential application of passive acoustic monitoring for soundscape characterization and long-term environmental monitoring in impacted regions.

An important contribution of this work is the demonstration that environmental acoustic information can also be used to validate and complement climatic measurements. Rainfall events detected by the tipping-bucket rain gauge generated corresponding broadband acoustic signatures in the spectrograms, enabling direct temporal correlation between climatic and acoustic datasets. In addition, periodic acoustic signatures generated by the camera actuation mechanism provided an independent temporal reference that facilitated verification of timestamp consistency among sensing modalities within the proposed architecture.

The climatic measurements revealed consistent microclimatic patterns associated with local environmental dynamics. The delayed thermal response observed in soil temperature measurements relative to atmospheric temperature variations reflects soil thermal inertia processes commonly observed in natural ecosystems. Similarly, inverse relationships between air temperature and relative humidity were consistently observed during rainfall events and nighttime periods, demonstrating the reliability of the acquired climatic data under long-term outdoor operation.

From an operational perspective, the long-term deployment of nine monitoring stations under real environmental conditions demonstrated the robustness of the proposed architecture. Despite exposure to humidity, insects, vegetation growth, intense solar radiation, and wildlife interaction, most stations remained operational throughout the monitoring campaign. During approximately 18 months of deployment, only one station was lost due to theft, while the remaining stations continued operating despite prolonged exposure to adverse environmental conditions. These results indicate that the proposed design achieved satisfactory reliability for remote environmental monitoring applications.

The deployment campaign also revealed practical factors affecting long-term energy availability. Vegetation growth surrounding some monitoring stations and seasonal variations in solar incidence gradually reduced the amount of solar radiation reaching the photovoltaic panels. These observations highlighted the importance of installation-site selection, periodic vegetation management, and regular maintenance procedures to ensure reliable autonomous operation over extended monitoring periods.

The field deployment also revealed important practical challenges associated with long-term autonomous environmental monitoring. Biological interactions, particularly ant infestations, caused damage to electronic components and highlighted the importance of enclosure sealing, preventive maintenance, and robust environmental protection strategies. Additional issues, including conventional microSD card failures and temporary reductions in energy harvesting capacity were also observed during the monitoring campaign. The replacement of conventional storage media with High Endurance microSD cards substantially improved storage reliability during continuous operation. Such observations are particularly relevant because many environmental monitoring studies report only controlled laboratory evaluations rather than prolonged field operation under adverse outdoor conditions. Several of these challenges would not have been identified during short-term laboratory experiments and were readily identified only after prolonged field deployment.

Compared with related environmental monitoring systems presented in Table 1, the proposed VISW Station combines climatic, acoustic, and image-based sensing within a single low-cost architecture capable of fully offline operation during extended deployments. The platform enabled complementary analysis involving vegetation dynamics and rainfall patterns, acoustic signatures associated with biological and climatic events, and interactions among multiple climatic variables.

The three sensing modalities were not jointly analyzed within a single multimodal analytical framework. Complementary analyses revealed multiple relationships among sensing modalities. Vegetation dynamics were evaluated in relation to rainfall patterns, acoustic spectrograms were interpreted together with biological and rainfall-related sound events, and climatic variables were jointly analyzed to investigate interactions among air temperature, soil temperature, relative humidity, and precipitation. These complementary analyses demonstrate the potential of the VISW Station for multimodal environmental monitoring and ecosystem assessment.

The proposed system currently relies on local data storage, requiring periodic field visits for data retrieval. In addition, the vegetation analysis performed in this study combined visual interpretation with ExG-derived GVI measurements and statistical correlation analysis. However, the approach remains limited to RGB-based vegetation indices and does not provide the spectral precision achievable with multispectral or hyperspectral sensors. Similarly, the acoustic analysis focused on soundscape characterization and environmental event identification without automated species recognition or taxonomic validation.

Future work will focus on the integration of real-time communication modules, enabling remote data transmission and near real-time environmental monitoring. Additional sensing modalities, including atmospheric pressure, solar radiation, wind speed, and water quality sensors, may also be incorporated into the platform. Furthermore, future developments may explore the implementation of edge artificial intelligence techniques for automated acoustic event detection, species classification, anomaly detection, and image-based environmental analysis directly on embedded hardware platforms.

## 5. Conclusions

This work presented the development and long-term field deployment of the VISW Station, a low-cost multimodal environmental monitoring system designed for autonomous operation in remote and post-disaster environments. Unlike conventional environmental monitoring approaches that typically focus on isolated sensing modalities or depend on communication infrastructure, the proposed platform successfully integrated climatic sensing, passive acoustic monitoring, and multispectral image acquisition into a fully autonomous edge-based architecture capable of continuous long-term operation in fully offline conditions.

The obtained results demonstrate the potential of multimodal environmental monitoring for capturing seasonal climatic variations, vegetation dynamics, and environmental acoustic activity under real field conditions. Relationships between rainfall patterns, vegetation recovery, and biological acoustic activity were successfully identified throughout the monitoring campaign, highlighting the importance of combining climatic, visual, and acoustic datasets for ecosystem analysis.

Long-term field deployment further demonstrated the operational robustness of the proposed architecture under adverse environmental conditions, including prolonged exposure to humidity, insects, vegetation growth, and outdoor environmental stressors. The low-cost design, reduced infrastructure requirements, and modular architecture indicate strong potential for scalable environmental monitoring applications in remote ecosystems and environmentally impacted regions.

Particularly in the context of the Fundão dam collapse, one of the largest environmental disasters in Brazil’s history, the proposed system demonstrated the feasibility of long-term autonomous monitoring for ecosystem recovery assessment in severely impacted environments. The generated multimodal dataset provides valuable information for future ecological studies, biodiversity monitoring, and environmental recovery analysis.

Overall, the Manna-VISW station offers a practical, flexible, and scalable solution for long-term environmental monitoring under real-world conditions. Future developments will focus on real-time communication, additional sensing modalities, and edge artificial intelligence for automated environmental analysis.

## Figures and Tables

**Table 1:** Comparison of selected environmental monitoring systems and the proposed VISW Station.

Reference	Audio	Imaging	Weather	Solar Powered	Field Deployment
Richardson et al. (2018) [14]	–	✓	–	NR	Multi-year PhenoCam network
Sethi et al. (2018) [6]	✓	✓	–	✓	12 months
Bernardes et al. (2023) [2]	–	–	✓	✓	30-day disaster-monitoring validation
Emvoliadis et al. (2024) [18]	✓	–	–	NR	Prototype evaluation
Ramesh et al. (2023) [19]	✓	–	–	NR	Seasonal restoration monitoring campaign
VISW Station	✓	✓	✓	✓	18 months

NR: Not reported in the corresponding publication.

**Table 2:** Geographic coordinates and sampling locations of the environmental monitoring stations installed along the Gualaxo do Norte and Doce river basins.

Station Code	Sampling Location	Latitude	Longitude
S1	Nova Bento Rodrigues	−20.2845	−43.4533
S2	Bufalo	−20.2399	−43.3302
S3	Paracatu de Baixo	−20.2999	−43.2315
S4	Areal	−20.0159	−42.7422
S5	Pousada	−20.0078	−42.7690
S6	Ipatinga 1	−19.5755	−42.5200
S7	Ipatinga 2	−19.6976	−42.4956
S8	Conselheiro Pena	−19.1706	−41.4420
S9	Aimorés	−19.3919	−41.1329

**Table 3:** GPIO connections and communication interfaces employed in the proposed environmental monitoring station architecture.

Module	Interface	GPIO	Physical Pin	Function
DHT22	Digital GPIO	GPIO 6	Pin 31	Air temperature and humidity
DS18B20	1-Wire	GPIO 5	Pin 29	Soil temperature
Rain Gauge	Digital Interrupt	GPIO 26	Pin 37	Rainfall pulse detection
L298N IN1	Digital GPIO	GPIO 8	Pin 24	RGB filter actuation
L298N IN2	Digital GPIO	GPIO 7	Pin 26	NOIR filter actuation
INMP441	I2S	GPIO 18/19/20	Pins 12/35/38	Environmental acoustic acquisition

**Table 4:** Bill of Materials (BOM) and approximate acquisition costs of the VISW Station.

Category	Description/Model	Qty	Cost (BRL)
Enclosure	Waterproof enclosure (102 × 102 × 55 mm, IP65)	1	25.00
Enclosure	Waterproof enclosure (260 × 110 × 70 mm, IP65)	1	25.00
Processing and Storage	Raspberry Pi Zero 2 W	1	250.00
Processing and Storage	High-Endurance microSD card	1	150.00
Imaging System	OV5647 with IR-CUT filter	1	100.00
Imaging System	L298N Mini driver module	1	5.00
Acoustic Monitoring	INMP441 MEMS microphone	1	35.00
Environmental Sensing	Rain gauge, meteorological shield, and DHT22 sensor assembly	1	500.00
Environmental Sensing	DS18B20 soil temperature sensor	1	25.00
Power Supply	Li-ion battery pack (3S, 15 Ah)	1	350.00
Power Supply	MPPT solar charge controller	1	150.00
Power Supply	Photovoltaic panel (10–30 W)	1	160.00
Mechanical Structure	Steel and aluminum mounting structure, brackets, and support elements	1	80.00
Assembly Materials	Wiring, connectors, cable glands, solder, and miscellaneous installation materials	1	40.00
Total Estimated Cost			1895.00

**Table 5:** Estimated VISW Station storage autonomy considering continuous multimodal data acquisition.

microSD Capacity	Usable Storage	Estimated Autonomy (Days)	Estimated Autonomy (Months)
64 GB	55.6 GB	67	2.2
128 GB	115 GB	139	4.6
256 GB	234 GB	282	9.3

**Table 6:** Monthly rainfall and GVI values obtained from the Conselheiro Pena monitoring station.

Month	Rainfall (mm)	GVI (%)
Feb/25	24	52.68
Mar/25	48	49.41
Apr/25	105	38.21
May/25	6	38.26
Jun/25	9	19.55
Jul/25	0	2.07
Aug/25	13	0.06
Sep/25	15	0.02
Oct/25	48	0.02
Nov/25	32	30.18
Dec/25	124	35.78
Jan/26	200	47.83
Feb/26	286	51.41

**Table 7:** Deployment schedule of the VISW monitoring stations.

Station	Location	Installation Date
S1	Nova Bento Rodrigues	September/2024
S2	Bufalo	September/2024
S3	Paracatu de Baixo	October/2024
S4	Areal	October/2024
S5	Pousada	October/2024
S6	Ipatinga 1	December/2024
S7	Ipatinga 2	December/2024
S8	Conselheiro Pena	February/2025
S9	Aimorés	February/2025

**Table 8:** Total amount of data collected by each VISW station during the monitoring campaign.

Station	Total Data (GB)
Nova Bento Rodrigues	152.10
bufalo	96.36
Paracatu de Baixo	86.16
Areal	281.82
Pousada	287.02
Ipatinga 1	129.78
Ipatinga 2	279.27
Conselheiro Pena	276.00
Aimorés	198.60
Total	1787.11

## Data Availability

The multimodal environmental datasets generated during the current study, including climatic, acoustic, and image-based data, are available from the corresponding author upon reasonable request.

## Abbreviations

The following abbreviations are used in this manuscript:

ACI	Acoustic Complexity Index
BOM	Bill of Materials
CMOS	Complementary Metal-Oxide-Semiconductor
CSI	Camera Serial Interface
CSV	Comma-Separated Values
ENT	Entropy
EVN	Acoustic Events
ExG	Excess Green Index
GPIO	General Purpose Input/Output
GVI	Green View Index
HybridExG	Hybrid Excess Green Index
HybridVI	Hybrid Vegetation Index
I2S	Inter-IC Sound
IoT	Internet of Things
IR	Infrared
IR-CUT	Infrared Cut Filter
LDR	Light-Dependent Resistor
LEEB	Ecology and Biodiversity Research Group
MANNA	Manna Research Group
MEMS	Microelectromechanical Systems
MPPT	Maximum Power Point Tracking
NDVI	Normalized Difference Vegetation Index
NIR	Near-Infrared
NOIR	No Infrared Filter
OGG	Ogg multimedia container format
PAM	Passive Acoustic Monitoring
QUT	Queensland University of Technology
RGB	Red, Green and Blue
ROI	Region of Interest
SSH	Secure Shell
STFT	Short-Time Fourier Transform
UEM	Universidade Estadual de Maringá
UFMG	Universidade Federal de Minas Gerais
VARI	Visible Atmospherically Resistant Index
VISW	Verona Image, Sound, and Weather

## Appendix A

**Table A1:** Main configuration parameters employed for ecoacoustic analysis and false-color spectrogram generation using the QUT Ecoacoustics Analysis Program.

Parameter	Value	Description
SegmentDuration	60 s	Audio segmentation duration
SegmentOverlap	0 s	Segment overlap
IndexCalculationDuration	60 s	Time interval used for index calculation
BgNoiseNeighborhood	5 s	Background noise estimation neighborhood
ResampleRate	24,000 Hz	Audio resampling frequency
FrameLength	512 samples	FFT frame length
LowFreqBound	1000 Hz	Lower bound of mid-frequency band
MidFreqBound	8000 Hz	Upper bound of mid-frequency analysis band
HighFreqBound	11,000 Hz	Maximum analyzed frequency
FrequencyScale	Linear	Spectral frequency scale
False-color indices	ACI–ENT–EVN	RGB channel assignment
EventThreshold	0.2	Acoustic event detection threshold

## References

[1] Morchid A., Marhoun M., El Alami R., Boukili B. (2024). Intelligent Detection for Sustainable Agriculture: A Review of IoT-Based Embedded Systems, Cloud Platforms, DL, and ML for Plant Disease Detection. Multimed. Tools Appl..

[2] Bernardes G.F.L.R., Ishibashi R., Ivo A.A.S., Rosset V., Kimura B.Y.L. (2023). Prototyping Low-Cost Automatic Weather Stations for Natural Disaster Monitoring. Digit. Commun. Netw..

[3] Reis M.J.C.S. (2026). Low-Power Embedded Sensor Node for Real-Time Environmental Monitoring with On-Board Machine-Learning Inference. Sensors.

[4] Carmo F.F., Kamino L.H.Y., Junior R.T., Campos I.C., Carmo F.F., Silvino G., Castro K.J.S.X., Mauro M.L., Rodrigues N.U.A., Miranda M.P.S. (2017). Fundão tailings dam failures: The environment tragedy of the largest technological disaster of Brazilian mining in global context. Perspect. Ecol. Conserv..

[5] Guerra M.B.B., Teaney B.T., Mount B.J., Asunskis D.J., Jordan B.T., Barker R.J., Santos E.E., Schaefer C.E.G.R. (2017). Post-catastrophe analysis of the Fundão tailings dam failure in the Doce River system, Southeast Brazil: Potentially toxic elements in affected soils. Water Air Soil Pollut..

[6] Sethi S.S., Ewers R.M., Jones N.S., Orme C.D.L., Picinali L. (2018). Robust, Real-Time and Autonomous Monitoring of Ecosystems with an Open, Low-Cost, Networked Device. Methods Ecol. Evol..

[7] Queiroz H.M., Nóbrega G.N., Ferreira T.O., Almeida L.S., Romero T.B., Santaella S.T., Bernardino A.F., Otero X.L. (2018). The Samarco mine tailing disaster: A possible time-bomb for heavy metals contamination?. Sci. Total Environ..

[8] Roostaei J., Wager Y.Z., Shi W., Dittrich T., Miller C., Gopalakrishnan K. (2023). IoT-Based Edge Computing (IoTEC) for Improved Environmental Monitoring. Sustain. Comput. Inform. Syst..

[9] Sueur J., Farina A. (2015). Ecoacoustics: The Ecological Investigation and Interpretation of Environmental Sound. Biosemiotics.

[10] Sugai L.S.M., Desjonquères C., Silva T.S.F., Llusia D. (2019). Terrestrial Passive Acoustic Monitoring: Review and Perspectives. BioScience.

[11] Stowell D., Wood M.D., Pamuła H., Stylianou Y., Glotin H. (2019). Automatic acoustic detection of birds through deep learning: The first Bird Audio Detection challenge. Methods Ecol. Evol..

[12] Towsey M., Znidersic E., Broken-Brow J., Indraswari K., Watson D.M., Phillips Y., Truskinger A., Roe P. (2018). Long-Duration, False-Colour Spectrograms for Detecting Species in Large Audio Data-Sets. J. Ecoacoust..

[13] Scarpelli M.D.A., Ribeiro M.C., Teixeira C.P. (2021). What does Atlantic Forest soundscapes can tell us about landscape?. Ecol. Indic..

[14] Richardson A.D., Hufkens K., Milliman T., Aubrecht D.M., Chen M., Gray J.M., Johnston M.R., Keenan T.F., Klosterman S.T., Kosmala M. (2018). Tracking vegetation phenology across diverse North American biomes using PhenoCam imagery. Sci. Data.

[15] Alberton B., Martin T.C.M., Rocha H.R., Richardson A.D., Moura M.S.B., Torres R.S., Morellato L.P.C. (2023). Relationship between tropical leaf phenology and ecosystem productivity using phenocameras. Front. Environ. Sci..

[16] Zeng Y., Hao D., Huete A., Dechant B., Berry J., Chen J.M., Joiner J., Frankenberg C. (2022). Optical vegetation indices for monitoring terrestrial ecosystems globally. Nat. Rev. Earth Environ..

[17] Yan K., Gao S., Yan G., Ma X., Chen X., Zhu P., Li J., Gao S., Gastellu-Etchegorry J.-P., Myneni R.B. (2025). A global systematic review of the remote sensing vegetation indices. Int. J. Appl. Earth Obs. Geoinf..

[18] Emvoliadis A., Mitianoudis N., Taitzoglou A., Vrochidis S., Kompatsiaris I. (2024). Multimodal Environmental Sensing Using AI and IoT Solutions: A Cognitive Sound Analysis Perspective. Sensors.

[19] Ramesh V., Hariharan P., Akshay V.A., Choksi P., Khanwilkar S., DeFries R., Robin V.V. (2023). Using passive acoustic monitoring to examine the impacts of ecological restoration on faunal biodiversity in the Western Ghats. Biol. Conserv..

[20] Fernandes G.W., Goulart F.F., Ranieri B.D., Coelho M.S., Dales K., Boesche N., Bustamante M., Carvalho F.A., Carvalho D.C., Dirzo R. (2016). Deep into the mud: Ecological and socio-economic impacts of the dam breach in Mariana, Brazil. Nat. Conserv..

[21] Raspberry Pi Foundation Raspberry Pi Zero 2 W Specifications. https://www.raspberrypi.com/products/raspberry-pi-zero-2-w/.

[22] Hill A.P., Prince P., Piña Covarrubias E., Doncaster C.P., Snaddon J.L., Rogers A. (2018). AudioMoth: Evaluation of a smart open acoustic device for monitoring biodiversity and the environment. Methods Ecol. Evol..

[23] Sheng Z., Pfersich S., Eldridge A., Zhou J., Tian D., Leung V.C.M. (2019). Wireless Acoustic Sensor Networks and Edge Computing for Rapid Acoustic Monitoring. IEEE/CAA J. Autom. Sin..

[24] Oppenheim A.V., Schafer R.W. (2010). Discrete-Time Signal Processing.

[25] McLoughlin I., Pham L., Song Y., Miao X., Phan H., Cai P., Gu Q., Nan J., Song H., Soh D. (2026). Spectrogram Features for Audio and Speech Analysis. Appl. Sci..

[26] QUT Ecoacoustics Research Group Audio Analysis Software Package. https://github.com/QutEcoacoustics/audio-analysis.

[27] Towsey M., Wimmer J., Williamson I., Roe P. (2014). The Use of Acoustic Indices to Determine Avian Species Richness in Audio-Recordings of the Environment. Ecol. Inform..

[28] Alberton B., Torres R.d.S., Cancian L.F., Borges B.D., Almeida J., Mariano G.C., Santos J., Morellato L.P.C. (2017). Introducing digital cameras to monitor plant phenology in the tropics: Applications for conservation. Perspect. Ecol. Conserv..

[29] Woebbecke D.M., Meyer G.E., Von Bargen K., Mortensen D.A. (1995). Color Indices for Weed Identification under Various Soil, Residue, and Lighting Conditions. Trans. ASAE.

[30] Gitelson A.A., Kaufman Y.J., Stark R., Rundquist D. (2002). Novel Algorithms for Remote Estimation of Vegetation Fraction. Remote Sens. Environ..

[31] Aoki Y. (1991). Evaluation Methods for Landscapes with Greenery. Landsc. Res..

[32] Yang J., McBride J., Zhou J., Sun Z. (2009). Can You See Green? Assessing the Visibility of Urban Forests in Cities. Landsc. Urban Plan..

